# Mapping and Analyzing Ecosystem Services Hotspots and Coldspots for Sustainable Spatial Planning in the Greater Asmara Area, Eritrea

**DOI:** 10.1007/s00267-024-02078-x

**Published:** 2024-11-03

**Authors:** Blal Adem Esmail, Chiara Cortinovis, Davide Geneletti, Luis Inostroza, Riccardo Peters, Claudia Romelli, Isabel Schulze, Belula Tecle-Misghina, Medhane Teklemariam, Jingxia Wang, Christian Albert

**Affiliations:** 1 Institute of Geography, Ruhr University Bochum, Bochum, Germany; 2 GLOMOS - Center for Global Mountain Safeguard Research & Institute for Alpine Environment, Eurac Research, Bolzano, Italy; 3 Department of Civil, Environmental & Mechanical Engineering, University of Trento, Trento, Italy; 4 Mendel University in Brno, Faculty of Regional Development & International Studies, Brno, Czech Republic; 5 Universidad Autónoma de Chile, Santiago, Chile; 6 Poliedra—Politecnico di Milano, Milan, Italy; 7 Lincoln School of Design and Architecture, University of Lincoln, Lincoln, UK; 8 Asmara Heritage Project, Maekel region, Asmara, Eritrea; 9 Department of Urban Studies and Planning, The University of Sheffield, Western Bank, Sheffield, UK; 10 Leibniz Universität Hanover, Institute of Environmental Planning, Hanover, Germany

**Keywords:** East Africa, IPBES, Land cover change analysis, Matrix approach, Urban planning, Sustainable cities and communities

## Abstract

Rapid urbanization in African metropolises like the Greater Asmara Area, Eritrea, poses numerous environmental challenges, including soil sealing, loss of vegetation cover, threats to protected natural areas, and climate change, among others. Mapping and assessing ecosystem services, particularly analyzing their spatial and temporal distribution is crucial for sustainable spatial planning. This study aims at mapping and analyzing ecosystem services hotspots and coldspots dynamics in the Greater Asmara Area to identify recent trends and opportunities for enhancing ecosystem services supply. Utilizing remote sensing images, we produced land cover maps for 2009 and 2020 and mapped six ecosystem services through a lookup table approach. The study includes provisioning, regulating and maintenance, and cultural ecosystem services. We analyzed their spatio-temporal variations, identifying ecosystem services hotspots and coldspots and their changes over time. Results show that overall ecosystem services potential in the Greater Asmara Area remains low but stable, with some improvements. By 2020, areas with no ecosystem services potential decreased in southern regions like Gala Nefhi and Berik, and new hotspots and coldspots emerged in central Gala Nefhi. This pilot study demonstrates the feasibility and key challenges of the ecosystem services hotspots and coldspots approach for sustainable spatial planning in rapidly urbanizing African metropolitan regions. Despite limitations, the study offers valuable insights into ecosystem services potentials, and related hotspots and coldspots dynamics, raising awareness and paving the way for further research and application.

## Highlights

- Ecosystem services (ES) hotspot/cold spot analysis aids sustainable planning in rapidly urbanizing African cities.
- We mapped ES hotspot/cold spot dynamics in Greater Asmara Area, Eritrea, from 2009 to 2020.
- GAA’s ES potential is low but stable, showing some improvements over time.
- It is crucial to interpret observed ES cold/hotspot dynamics in GAA with caution.
- Our approach is replicable in other resource-scarce, rapidly urbanizing African cities.

## Introduction

Mapping and assessment of ecosystems and their services (MAES) is widely recognized as essential for achieving sustainable development (Geijzendorffer and Roche 2014; Geneletti et al. 2020a; Adem Esmail et al. 2023). MAES can significantly contribute to policy and decision-making processes at various levels, from increasing stakeholder awareness to guiding specific decisions (Posner et al. 2016). Ecosystem services (ES) knowledge can be used to create actionable policies, thereby enhancing human well-being alongside biodiversity and nature conservation (Mckenzie et al. 2014; Posner et al. 2016). Integrating ES assessments into spatial planning processes, including urban planning and environmental assessments such as strategic environmental assessments (SEA) and environmental impact assessments (EIA), has demonstrated considerable benefits such as the identification of ES trade-offs, the generation of baseline data about ES or the possibility to monitor and compare different planning alternatives (Geneletti 2013; Mckenzie et al. 2014; Rall et al. 2015; Albert et al. 2016; Cortinovis and Geneletti 2018; Longato et al. 2021).

While mapping individual ES helps to understand their distribution across the landscape, identifying hotspots and coldspots of ES provision can provide valuable support for sustainable spatial planning, among others to identify priority conservation areas (Mitchell et al. 2021). The definition of ES hotspots in the literature varies, with some authors using the term to refer to areas characterized by high levels of supply of a certain ES (Bai et al. 2011; Cimon-Morin et al. 2013). However, the term hotspots may also refer to areas with high levels of multiple ES provision, while coldspots are areas with low levels (García-Nieto et al. 2013; Geneletti et al. 2018). The latter are the definitions used in this study. Moreover, there exist various methods to identify ES hotspots, depending on the policy question to be addressed and the type of individual ES maps to be combined (Schröter and Remme 2016). Methods range from the simple overlapping of individual hotspots maps (García-Nieto et al. 2013; Peña et al. 2018), to more complex measures of occurrence such as intensity and richness (Plieninger et al. 2013), to statistical analysis techniques such as the Getis-Ord Gi* statistic (Bagstad et al. 2016). Geneletti and Cortinovis (2021) illustrate a real-world application at the city scale where a hotspots map summarizing the provision of seven ES was used to support the identification of key areas in the urban plan of Trento, Italy. Beyond single-time analyses, the study of hotspots and coldspots dynamics can reveal patterns of change in ES provision, supporting the identification of the underlying drivers (Li et al. 2016).

Pressing global challenges such as biodiversity loss, environmental degradation, and land cover change threaten the long-term integrity of ecosystems and the stability of ES provision. Anthropogenic land cover changes are the primary drivers of ES dynamics (MEA 2005; de Groot et al. 2012; Costanza et al. 2014; Diaz et al. 2019). Therefore, multi-temporal analyses of land cover change and related ES dynamics can reveal past trends in ES provision and recommend courses of action for its protection and enhancement, thereby increasing the benefits of MAES applications for planning (Lyu et al. 2021). Despite the recognized importance, few studies focus on the dynamic evolution of ES, and even fewer analyze changes in ES hotspots and coldspots. Furthermore, the distribution of studies is highly skewed, with the majority conducted in the Global North. This underrepresentation is particularly evident in Africa, despite the proven effectiveness of MAES in governance, evaluation (Azadi et al. 2012), and sustainable policy and decision-making (Adem Esmail et al. 2023).

In this study, we propose a MAES pilot to support sustainable planning in the rapidly urbanizing Greater Asmara Area (GAA) in Eritrea. This study aims at mapping and analyzing ES hotspots and coldspots dynamics in the GAA to identify recent trends and opportunities for enhancing ES potential. The GAA, the largest urban area in Eritrea and including a UNESCO World Heritage Site, houses 50–60% of the country’s population (Ministry of Public Works and BCEOM 2006; Ghebru et al. 2011). It faces risks from local urbanization, regional rural resource degradation, and global climate change impacts (MoLWE 2012a). This pilot study in the GAA seeks to raise awareness of these challenges and their effects on ecosystems and their services.

We map and assess six illustrative ES using land cover data from 2009 and 2020, obtained via remote sensing. To evaluate changes in ES supply potential, we employ the ES matrix approach by (Burkhard et al. 2010), a tier 1 MAES method suitable for data-scarce regions, as demonstrated in Eritrea and Kenya (Wangai et al. 2019; Adem Esmail et al. 2023). This analysis identifies recent trends and future land use opportunities by considering changes in ES hotspots and cold stops distribution across the GAA.

## Material and Methods

### The Study Area

Like many African countries, Eritrea relies heavily on natural capital, making it vulnerable to environmental challenges, such as droughts, which are exacerbated by climate change (MoLWE 2012a; Wangai et al. 2016; IPBES 2018). The country faces a variety of challenges, including low availability of arable land and water scarcity, which can significantly impact food security and overall human well-being. Studies suggest that the average temperatures in Eritrea could increase by up to 3.39°C by 2080 (Hunt et al. 2019). These environmental stressors underscore the urgent need for effective governance and planning strategies informed by ES knowledge, particularly in urban contexts.

**Fig. 1:**
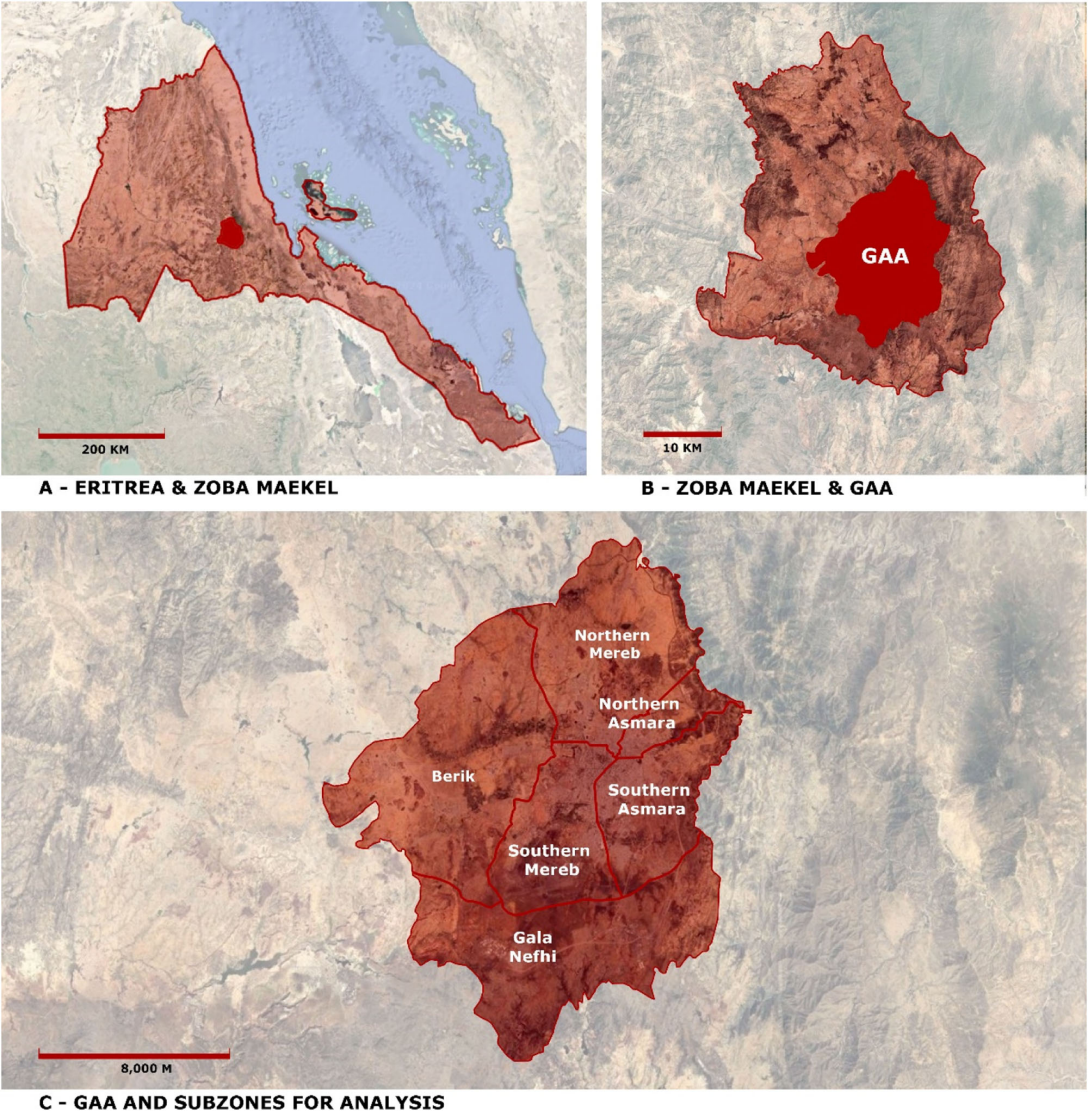
Study area location in Eritrea, in the Horn of Africa [**A**] and in the Maekel or Central region, one of the six administrative regions in the country [**B**]. The Greater Asmara Area (GAA) divided into six subzones for analysis based on the sub-regional boundaries in the Zoba Maekel [**C**]. NB. The subzones in the GAA are not official administrative areas; rather, they are analytical units for the present study only. (Sources: Google Earth, FAO, digitization of the SUDP)

As illustrated in Fig. 1, the GAA is situated in the Maekel region, which is part of the highlands where an estimated 50–60% of the country’s total population resides (Ministry of Public Works and BCEOM 2006; Ghebru et al. 2011). The GAA was initially delineated by *‘The Planning Committee’* in 1998, encompassing Asmara Proper with its UNESCO World Heritage Site and several satellite villages. It was subsequently established in 2005 by the *Strategic Urban Development Plan (SUDP)* until 2025, as part of the *Asmara Infrastructure Development Study* (Tecle-Misghina 2014). Spanning from 15°13 N to 15°25 N and 38°48 to 38°57E, the landscape of the GAA can be categorized into three distinct zones: a central urban zone, a sizable peri-urban transition area, and an agricultural hinterland. In this study, we have considered formal administrative areas, namely Zoba Maekel, which defines subzones, and GAA, which defines metropolitan boundaries. We have combined these two formal administrative boundaries to facilitate the identification of administrative zones and respective sub-zones, which are also meaningful for our analysis.

Despite Eritrea’s overall climatic challenges, the highlands surrounding Asmara benefit from relatively favorable conditions. Situated at an altitude of ~2300 m on the plateau, the region experiences a total annual rainfall of 519 mm (recorded during the period of 1961–1990; (Deutscehr Wetterdienst, 2021), primarily concentrated during the rainy season of July (175 mm) and August (156 mm), referred to as “*kremti*” rain (Deutscehr Wetterdienst, 2021). Additionally, there is slightly higher rainfall in the spring months of April to June (known as “*asmera*” rain), ranging from 33 to 41 mm. The temperatures remain consistently around an average of 15.6 °C (recorded during the period of 1961–73 to 1990; (Deutscehr Wetterdienst, 2021), contributing to a dry climate. Given the favorable conditions in the highlands, the preservation of high-quality agricultural areas becomes even more crucial.

The seasonal patterns of rainfall significantly impact vegetation cycles and agricultural practices, particularly in the context of subsistence-based farming reliant on rainfall. Subsistence agriculture stands as a prominent feature of the landscape, with up to 70% of the Eritrean population depending on it for sustenance (Ghebru et al. 2011). While the tertiary and industrial sectors in the GAA may reduce the overall agricultural significance of the study area, agricultural land remains predominant, alongside scrubland, fallow land, and grazing areas crucial for pastoralism (Ministry of Public Works and BCEOM 2006). Nationally, ensuring food security remains critical, as Eritrea can only partially meet its nutritional requirements. This challenge extends to Asmara, as highlighted by the SUDP (Ministry of Public Works and BCEOM 2006), although the precise extent remains uncertain.

The socio-ecological challenges facing the GAA are multifaceted and significant. Historically, Eritrea has experienced a drastic reduction in forest coverage, declining from 30% to a mere 1%, largely due to historical agricultural practices, deforestation for construction purposes, and fuel consumption (MoLWE 2012a). Others propose that the forest cover in the country at the beginning of the 20th century was between 7 and 10 percent (Orioli and Molla, 2022). Still, this loss of forest cover has contributed to erosion, overgrazing, and desertification (MoLWE 2012a). Our study area follows the trends of nearby sub-Saharan regions, where anthropogenic disturbances are the main driving factor of the observed loss of vegetation cover (Zewdie et al. 2017). The rapid pace of urbanization in recent decades has exacerbated these challenges, leading to a four-fold increase in urban land between 1989 and 2009 within the GAA, at the expense of agricultural capacity and forested areas (Tewolde and Cabral 2011). As highlighted in the SUDP, the expansion of urban areas and the transformation of surrounding agricultural landscapes have introduced complex and often conflicting objectives for policymakers and planners. Balancing the needs of urban development with the preservation of local ecosystems and their services is paramount (Elmqvist et al. 2016; Geneletti et al. 2020b). Yet, addressing these ecological challenges requires a comprehensive understanding of the dynamics at play and a concerted effort to implement sustainable management practices that promote environmental conservation while accommodating urban growth.

### Methodology for MAES of the Greater Asmara Area

#### A 3-step approach for MAES

**Fig. 2:**
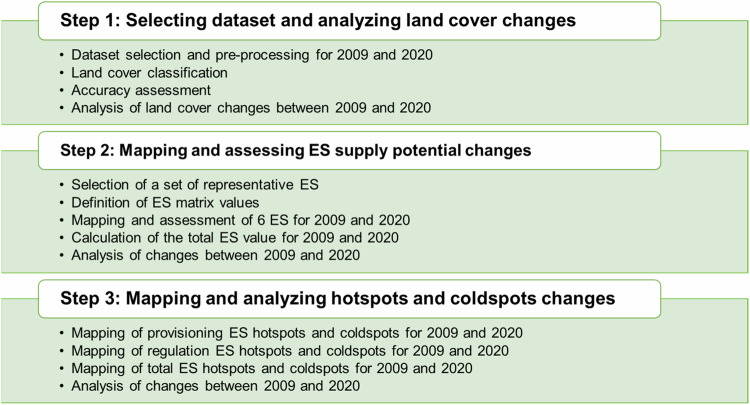
Steps of the methods

The research design comprises three main steps (Fig. 2). Firstly, we analyze land cover changes between 2009 and 2020 using remote sensing data. Secondly, we map and assess the potential supply of ES and calculate changes between 2009 and 2020. Finally, we produce hotspots and coldspots maps to analyze the changes between 2009 and 2020 and draw conclusions for the further spatial development of the GAA.

#### Step 1: Selecting dataset and analyzing land cover changes

Global datasets on land use and cover, such as those released by ESRI (Karra et al. 2021) and (Zhang et al. 2024), provide a comprehensive overview of land cover patterns across the world. However, upon a detailed examination of the land cover data in the GAA, noticeable uncertainties emerge, particularly in the classification and mixture of built-up areas and bare ground. Therefore, we produced our own land cover dataset for the GAA, following four key phases: dataset selection and pre-processing, land cover and use classification, accuracy assessment and land cover changes analysis.

We selected valid Landsat datasets for 2009 and 2020 to analyze land cover changes, with Landsat-5 carrying a multispectral scanner with a 30-meter resolution for 2009, and Landsat-8 offering improved spectral resolution and sensitivity for 2020. The classification was based on the *Africover* classification scheme by the Food and Agriculture Organization of the United Nations (FAO 1997), which identified eight land cover classes, ranging from urban/artificial areas to water bodies. This land cover scheme was selected due to its suitability for ES mapping and assessment in Eritrea, including Asmara (Adem Esmail et al. 2023). To implement this scheme, a Random Forest (FR) classifier was employed, with different classes examined through multiple bands such as red, green, blue and near-infrared and false color infrared compositions. Training points were employed to identify the eight classes: urban, water, forest, rainfed and irrigated agriculture, shrubs, bare land, and pasture.

Furthermore, to remove the “salt-and-pepper” noise that persists after classification, post-processing techniques were applied (Wang et al. 2019), such as post-classification smoothing filter with a kernel size of 2.5. The results were subject to manual review for both study periods to ensure the reflection of changes in land cover, such as the disappearance of water bodies.

All data was prepared and clipped in the same reference system (WGS_1984_UTM-Zone 37N). The land cover maps for 2009 and 2020, created in Google Earth Engine, were imported into ArcMap 10.5.1 for further analysis (see Figs. A1 and A2 in the Supplementary Material). The analysis of land cover changes at the regional and sub-regional levels between 2009 and 2020 was conducted in accordance with the methodology outlined by the ESCAP Statistics Division. This involved the generation of a transition matrix and a table of percent land cover changes relative to 2009. Zonal statistics were also calculated at both levels.

#### Step 2: Mapping and assessing ES supply potential changes

##### Selection of a set of representative ES

To represent the multifaceted development of the peri-urban area around Asmara and the diverse needs of the region, we selected a comprehensive range of ES. The selection criteria include representation of different sections of the Common International Classification of Ecosystem Services (CICES V4.3) and relevance for the case study region, as identified by expert co-authors with knowledge of the local context (BAE, BTM, MTM). The selection thus covers the domains of food, and water security (*ES1—Cultivated terrestrial plants*, *ES2—Animals reared for nutritional purposes*, and *ES3- Control of Erosion Rates*), local climate regulation *(ES4 - Regulation of temperature and humidity*), biodiversity conservation (*ES5 - Maintaining nursery populations and habitats*), and nature-based recreation (*ES6 - Characteristics of living systems that enable activities promoting health recuperation or enjoyment through active or immersive interactions*), thereby addressing some of the pressing socio-ecological challenges in the GAA.

##### Defining ES potential values and mapping

This study follows the tiered approach to ES mapping and assessment, as proposed by Grêt-Regamey et al. (2015). The tiers represent different levels of data integration and modeling complexity. Our study is associated with the coarsest level of analysis (level 1, as described by (Burkhard et al. 2010), where the assessment of ES is mainly based on land cover types. Although this coarseness limits its usefulness in detailed land use decisions, necessitating supplementary fieldwork and site-specific assessments, it is adequate for estimating the potential supply of ES on a regional scale and their spatial and temporal distribution (Montoya-Tangarife et al. 2017).

The ES potential for different land cover classes was mainly derived from a study conducted at the national level in Eritrea by Adem Esmail et al. (2023), where minimum, maximum, and average values were obtained based on a targeted literature review. For this study, average values were adopted to avoid extremes that were too high or too low. Additionally, a study by Augstburger et al. (2018), which specifically refers to local agroecosystems, was also taken into consideration to refine some of the values. The final ES matrix is presented in Table 1.

**Table 1:** Ecosystem Service Matrix potential on a 0 to 5 scale. The ecosystem services according to CICES v4.3 (Values after (Augstburger et al. 2018; Adem Esmail et al. 2023)

	ES Section	ES Class	Water	Forest	Irrigated Agriculture	Rainfed Agriculture	Shrubs	Bareland	Urban/Artificial	Grazingland
**ES1**	Provisioning	Cultivated terrestrial plants grown for nutritional purposes (1.1.1.1)	0	0.5	5	3.5	0	0	0	0
**ES2**	Provisioning	Animals reared for nutritional purposes (1.1.3.1)	0	1.5	2	2	1	0	0	4
**ES3**	Regulation & Maintenance	Control of erosion rates (2.2.1.1)	1	4.5	3.5	3	3	0,5	0	3
**ES4**	Regulation & Maintenance	Regulation of temperature and humidity, including ventilation and transpiration (2.2.6.2)	1	4	1	1	1	0	0	1
**ES5**	Regulation & Maintenance	Maintaining nursery populations and habitats, including gene pool protection (2.2.2.3)	4	3.5	3	3	2.5	0.5	0	0
**ES6**	Cultural	Characteristics of living systems that enable activities promoting health, recuperation or enjoyment through passive or observational interactions (3.1.1.1)	4	3	1	1	1	0.5	0	1.5

##### Mapping and assessment of selected ES and their change

The six ES were mapped for the years 2009 and 2020 based on the land cover data and the ES potential values presented in Table 1. Zonal statistical analysis was conducted with consideration of the sub-regional administrative boundaries within the GAA, thus facilitating a comparison of changes between the years 2009 and 2020. Accordingly, the trend was characterized as follows: “increasing/decreasing” (with a delta greater than ±0.4), “moderately increasing/decreasing” (with a delta between ±0.2 and ±0.4), and “stable” (a delta between −0.2 and 0.2).

#### Step 3: Mapping hotspots and coldspots and their dynamics

The analysis was performed using “Optimised Hot-Spot Tool” in ArcGis Pro ©. The tool calculates hotspots and coldspots based on the Getis-Ord* statistic, i.e. it identifies areas in the input map where high and low values are significantly clustered. It evaluates the characteristics of the input feature class to produce optimal results without predefining a search radius.

Three maps were created for each year: two maps for the provisioning ES (ES1 and ES2), two maps for regulation and maintenance ES (ES3, ES4, and ES5), and two maps for the total ES potential. The input maps for the hotspots analysis, were calculated as an arithmetic mean. No hotspots map has been created for “Recreation” as an individual cultural ES, as it only depends on Water and Forests according to the ES matrix. Finally, the changes in terms of hotspots and coldspots were analyzed, to understand the recent dynamics and provide recommendations for future spatial intervention options, including those for enhancing ES coldspots.

## Results

### Land cover changes

#### Accuracy assessment

The overall accuracy of the land cover classification was 0.79 in 2009 and 0.75 in 2020. The Kappa value, reflecting the agreement between observed and predicted classifications, dropped from 0.74 in 2009 to 0.67 in 2020. Of note, four classes (i.e. Irrigated agriculture, Rainfed agriculture, Shrubland, and Fallow Land showed very low producer accuracy (0–0.33) and consumer accuracy (0–0.5). For more details of the accuracy assessment results refer to Table A1 in the SM.

#### Changes in the Greater Asmara Area between 2009 and 2020

**Fig. 3:**
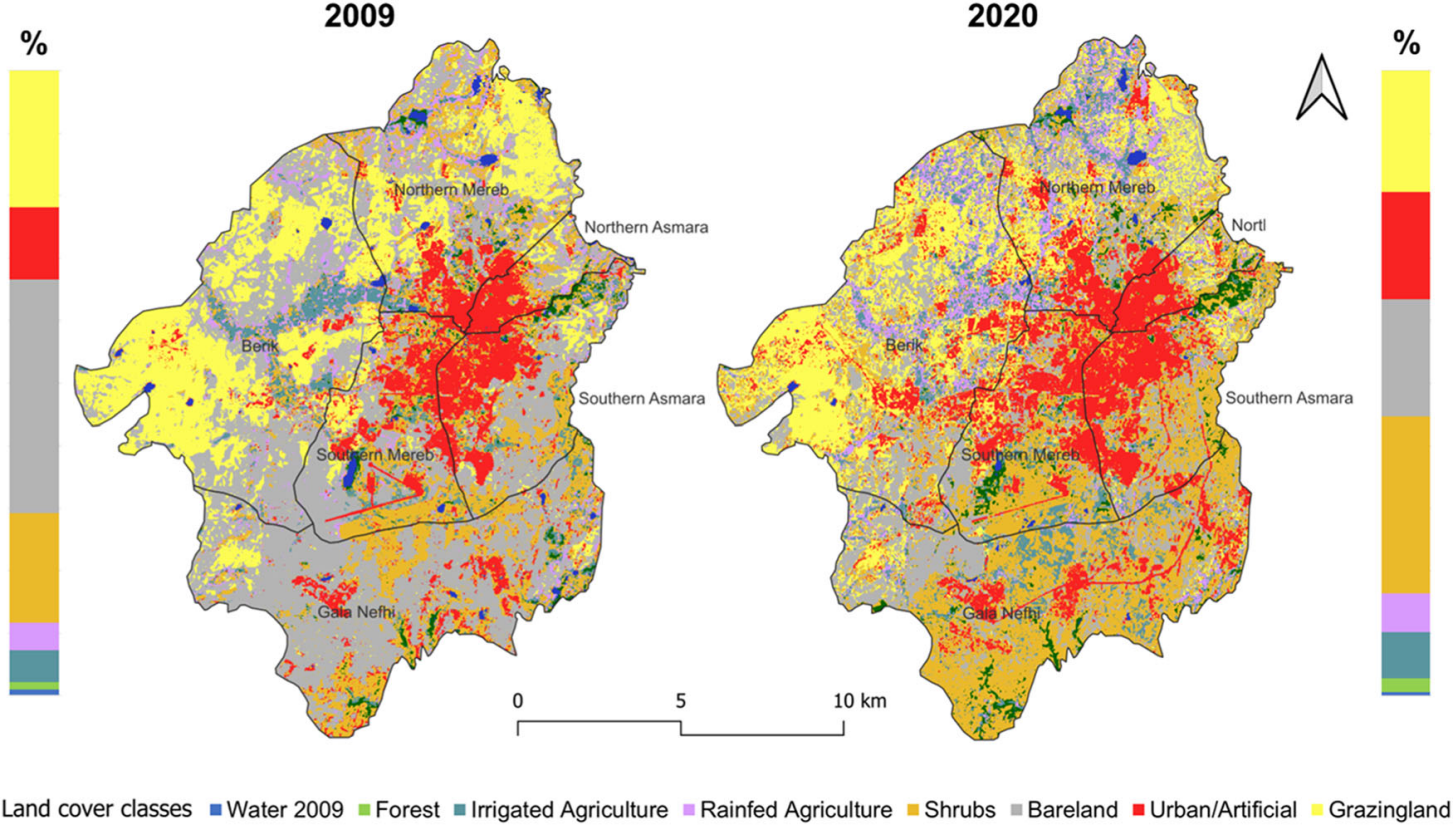
Comparison of land cover classifications for 2009 and 2020 and Proportions of classes for 2009 and 2020

Urbanization has intensified in the GAA (Fig. 3), with urban and artificial land cover expanding by notable 1179.3 ha (or 48.5%). Forested areas have also seen a remarkable increase of 225 ha (91%), while bare land has significantly decreased by 3917.4 hectares (a reduction of 49.8%). Grazing land has also experienced a decline, shrinking by 516.6 ha (−11.2%). On the agricultural front, both irrigated and rainfed farming has expanded, with increases of 486.9 ha (a 45.7% increase) and 374.4 ha (a 39.7% increase), respectively. For detailed analysis, please refer to Figs. A3 to A5 in the SM.

#### Subzones of the Greater Asmara Area—Changes Between 2009 and 2020

**Fig. 4:**
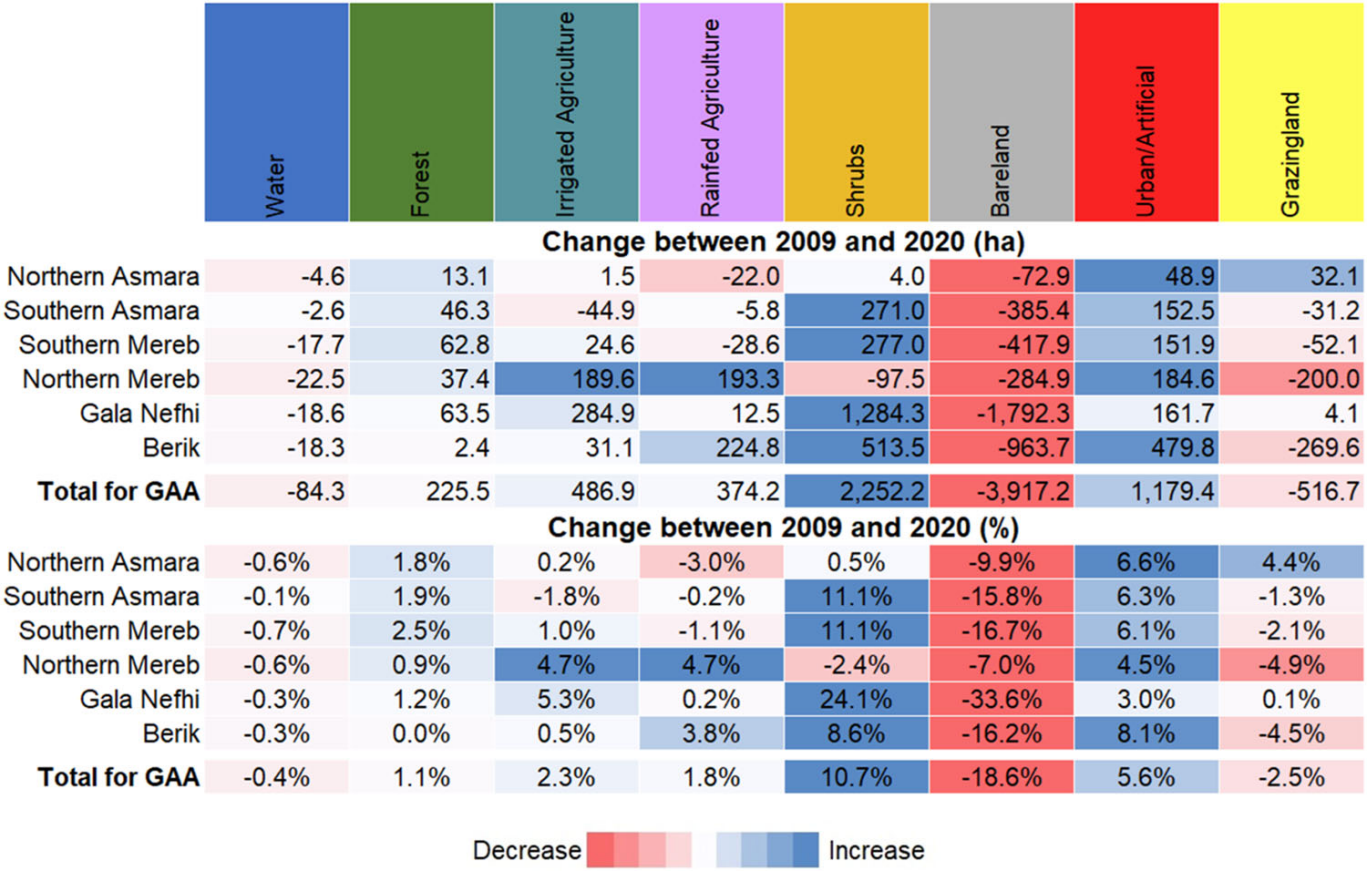
Sub-zonal and total land cover change in the GAA between 2009 and 2020 in absolute terms (ha) and percentage. Increases are shown in blue and decreases in red

Urbanization stands out prominently, notably in Berik and Northern Mereb, i.e. 479 ha and 185 ha, respectively (Fig. 4). In contrast, forested areas have increased steadily in Gala Nefhi, South Mereb, and South Asmara (64 ha, 63 ha, and 46.3 ha, respectively), indicating regeneration. Shifts in agriculture can be observed in the increased irrigated farming in Gala Nefhi and Northern Mereb (+284.9 ha and 189.6 ha, respectively), with declines in rainfed agriculture in Northern Asmara and Southern Mereb (22 ha and 28.2 ha, respectively).

Percentage changes reveal nuanced patterns: for instance, Northern Asmara had a slight decrease in water area (−0.6%) but an increase in forest cover (+1.8%). Southern Asmara experienced a decline in irrigated agriculture (−1.8%) but significant forest expansion (+1.9%). Southern Mereb witnessed decrease of rainfed agriculture (−1.1%) and more shrubland (+11.1%), while Northern Mereb observed increases in both irrigated and rainfed agriculture (+4.7%) alongside declines in bare land and grazing land. Gala Nefhi witnessed increased irrigated agriculture (+5.3%) and decreased bare land (−33.6%), while Berik saw a rise in rainfed agriculture (+3.8%) alongside declines in bare land and grazing land.

### ES Mapping and Assessment for 2009 and 2020

**Fig. 5:**
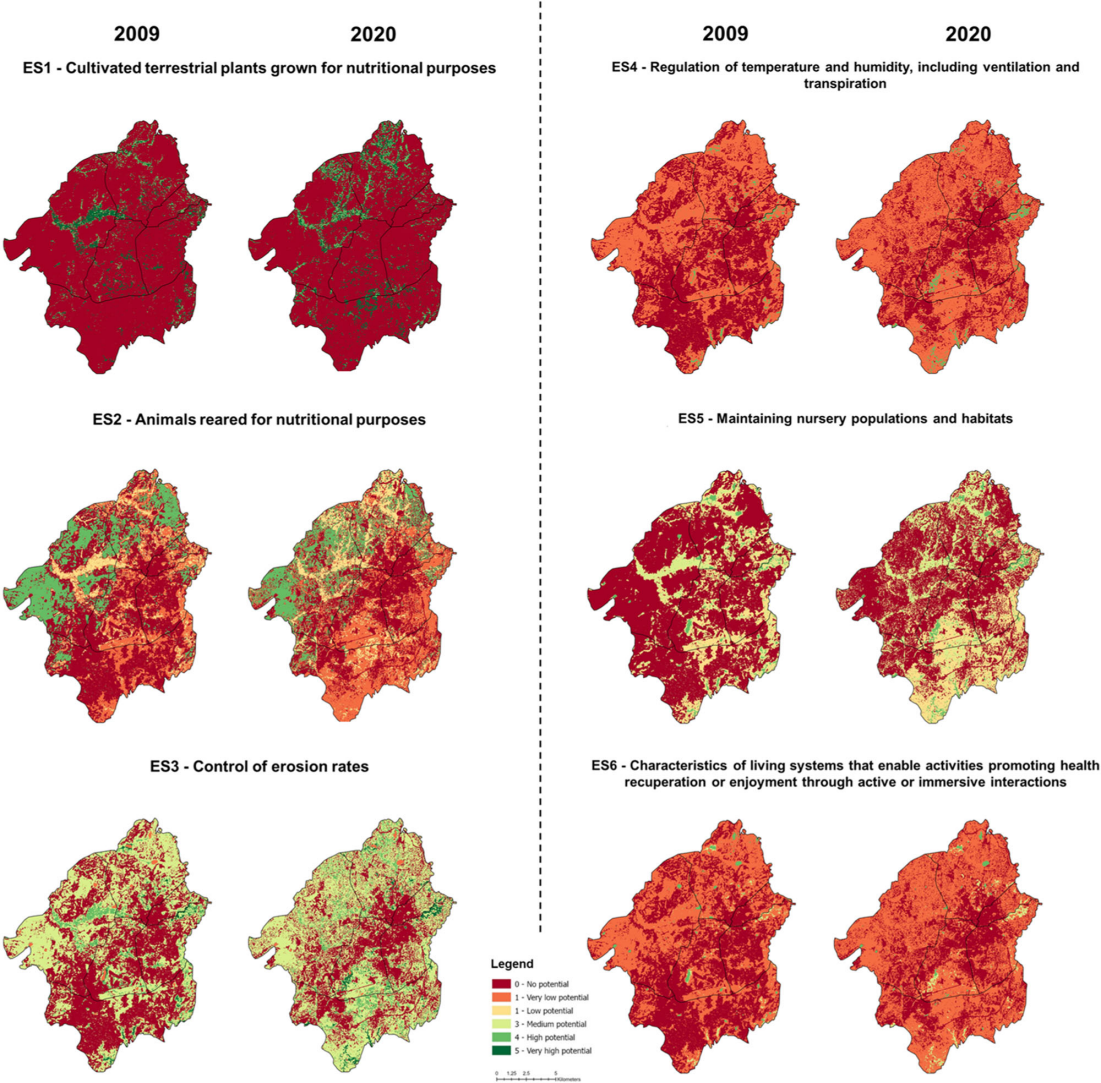
ES potential maps for the selected set of ES in the GAA for 2009 and 2020

The results indicate that the ES potential in the GAA is rather limited, with a maximum mean value of ES potential of 1.96 for erosion control (ES3) in 2020 (Fig. 5). This corresponds to “Low potential” according to the 0–5 scale of the Matrix approach (Burkhard et al. 2010). The GAA performs relatively better in terms of *ES3—Control of Erosion Rates* and *ES5 - Maintaining nursery populations and habitats*. While the worst potential is in terms of *ES1- Cultivated terrestrial plants* (0.57) and *ES4—Regulation of temperature and humidity* (0.69). Northern Mereb achieving the highest overall score (1.30), performs relatively better in erosion control (ES3) and maintaining nursery populations (ES5). Berik (1.27) and Gala Nefhi (1.16) also perform relatively well, showing similar scores in these same two ES. In contrast, Northern Asmara (0.87) exhibits the lowest overall performance. It is noteworthy that Berik exhibits relatively high performance in animal rearing (ES2) and erosion control (ES3), while Gala Nefhi performs relatively well in habitat maintenance (ES5).

**Fig. 6:**
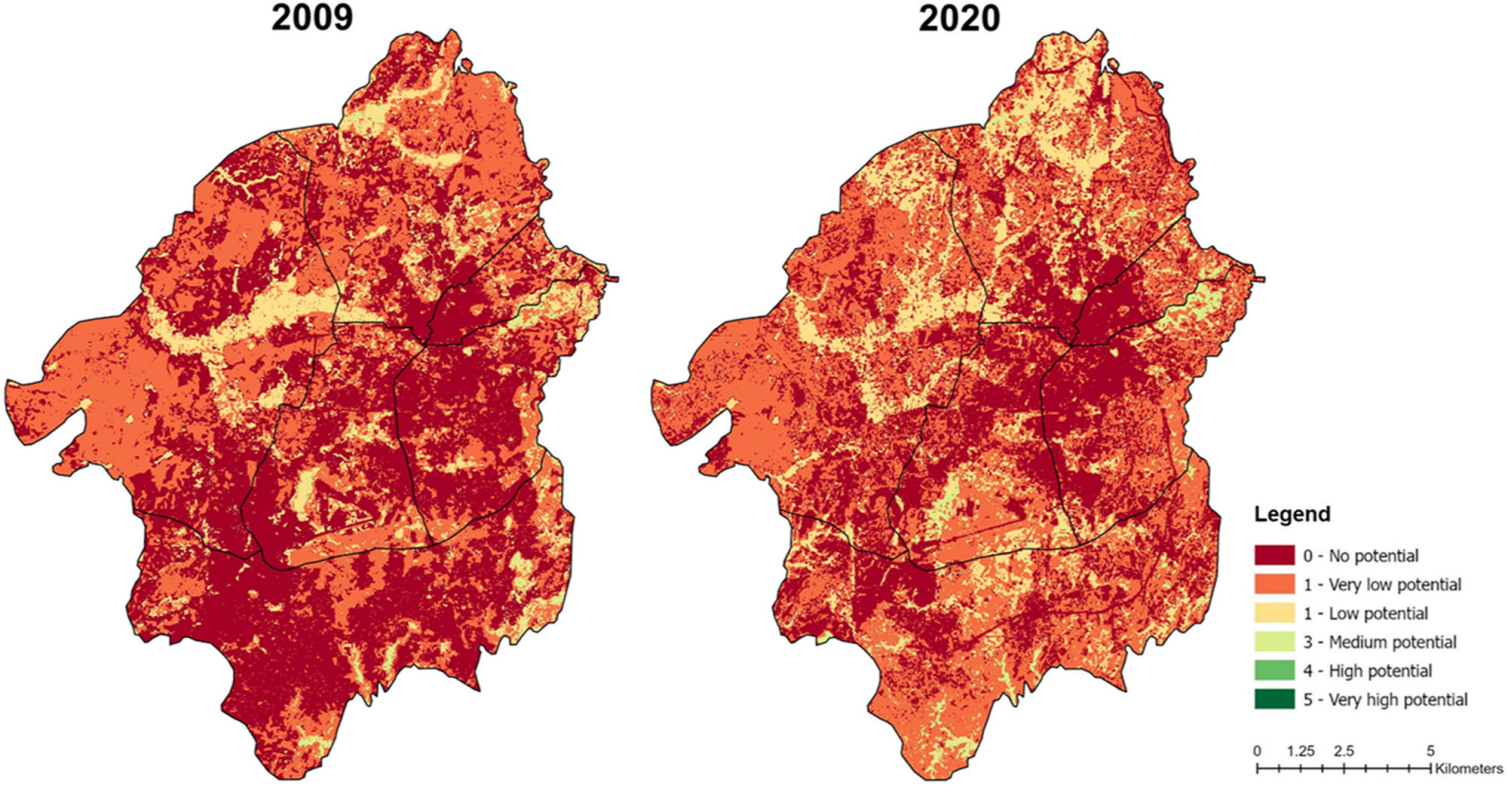
Total ES potential in the GAA for 2009 and 2020, calculated as the arithmetic mean of the six selected ES

Looking at the total ES potential in the GAA, the comparison between 2009 and 2020 confirms a low potential with some slight improvements (Fig. 6). There is a notable decrease of the areas with no potential (dark red) particularly in the southern regions (Gala Nefhi and Berik) and an increase of the areas with low potential (yellow) in the northern part of GAA (Northern Mereb and Berik).

**Fig. 7:**
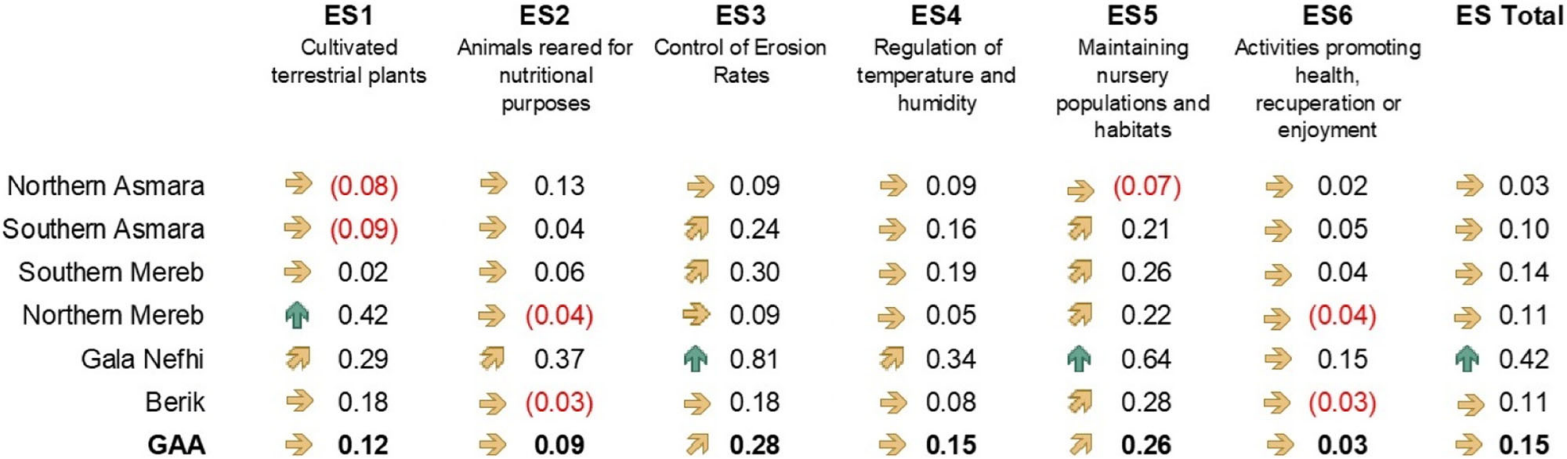
Mean values of ES potential in the sub-zones and their trends between 2009 and 2020. Green arrows (↑) highlight areas with increasing trend with a delta that is greater than 0.4. Upward arrows (↗) and downward arrows (↘) indicate moderate positive and negative trends, respectively, with delta between 0.2 and 0.4. Stable trends (→) represented by a horizontal arrow correspond to a delta between −0.2 and 0.2

Between 2009 and 2020, the ES potential in the GAA remained mostly stable, with notable improvements observed in *ES3—Control of erosion rates* (+028) and *ES5— Maintaining nursery populations and habitats* (+0.26)—see Fig. 7. Gala Nefhi stands out with increasing trends (delta greater than 0.4) in the total ES potential, particularly in ES5 and ES3. Northern Mereb shows a notable positive trend in cultivated terrestrial plants (ES1). Other regions, such as Northern and Southern Asmara, Southern Mereb, and Berik, exhibit a stable overall trend (delta between ±0.2). These MAES findings are described in more detail in the SM, including the individual ES potential maps and overall statistics (Figs. A6–A12).

### Mapping ES Hotspots and Coldspots Dynamics

**Fig. 8:**
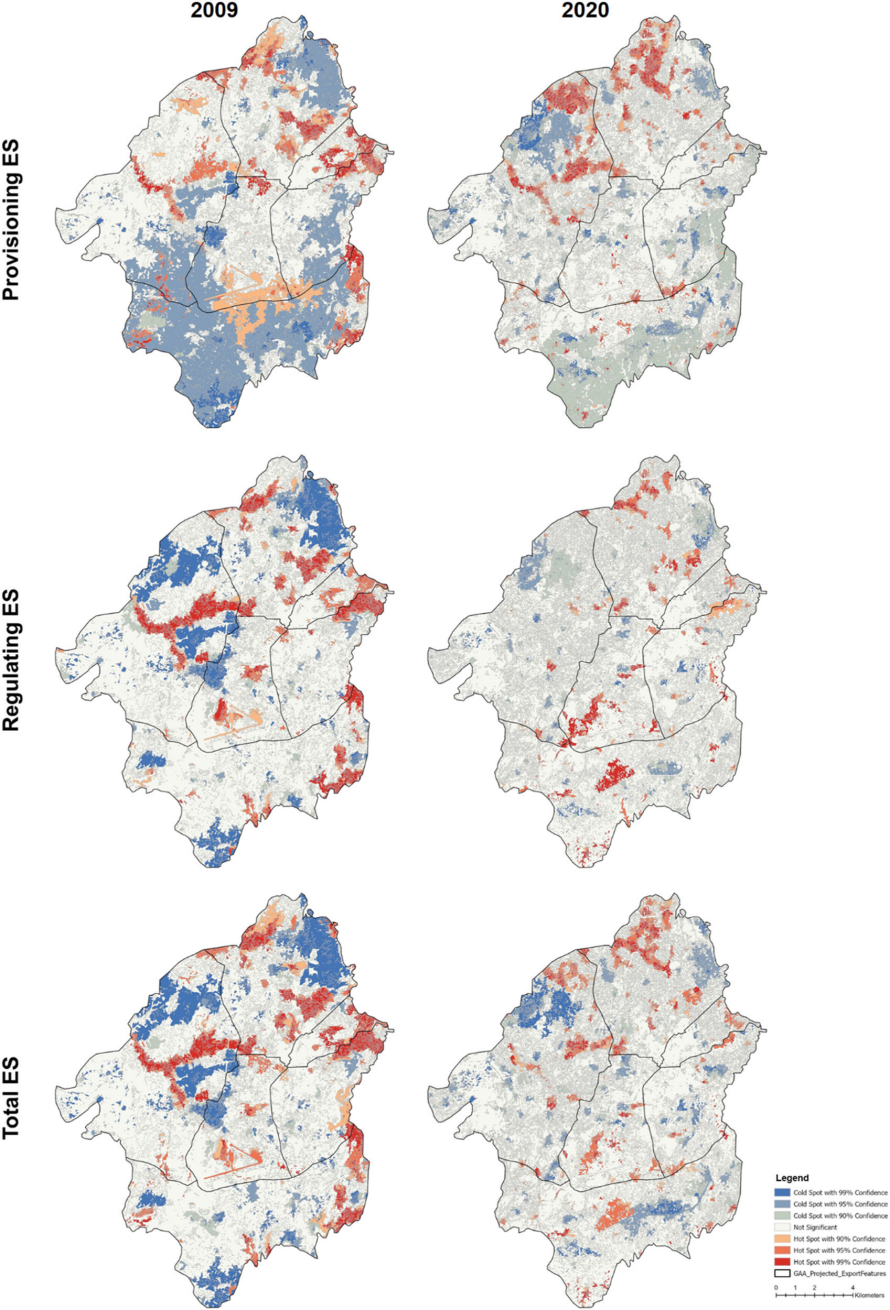
Changes in the Hot (red) and Cold (blue) Spots of the Provisioning, Regulation, and Maintenance, and Total ES potential between 2009 and 2020

Hotspots and coldspots of the provisioning, regulating and maintenance, and total ES potential for the year 2009 and 20020 are shown in Fig. 8. In 2009, the south-western regions (Gala Nefhi and Berik) predominantly featured significant coldspots (blue areas) for provisioning ES, indicating a concentration of areas with low agricultural and grazing potential. Hotspots (red areas) were sparse and scattered, mainly in the northeastern part. By 2020, there is a notable shift with an increase in hotspots, especially in the northern and northeastern regions (Northern Mereb and Berik), and a marked reduction in coldspots, reflecting improved provisioning capabilities and a lower clustering of areas with low potential.

Similarly, for regulating ES, 2009 saw widespread coldspots in the western and southern regions with fewer hotspots. However, by 2020, the majority of the previously identified coldspots had disappeared, along with some previously identified hotspots in Berik (see MaiBela floodplain) and Northern Asmara. In contrast, new hotspots emerged in the Gala Nefhi and Southern Mereb.

A similar trend is also seen in the hotspots analysis based on total ES potential. In 2009, the landscape was characterized by coldspots in the Northern Mereb, a major hotspot in the MaiBela floodplain in Berik, and the eastern parts of Northern Asmara and Gala Nefhi. On the other hand, in 2020, a notable reduction in the number of coldspots can be observed, except for the one Berik. Additionally, most previous hotspots had disappeared, with new ones emerging, such as in the center of Gala Nefhi.

## Discussion

### Strengths and Limitations of the Proposed Approach

The proposed methods for mapping land cover from remote-sensing data are adaptable to various future research objectives, resource availability, and database constraints. However, the importance of accurate land cover data in studies involving ES should be underscored, advocating for the consideration of higher-quality remote sensing images in forthcoming applications in the GAA. This is particularly relevant when comparing and distinguishing the land use classes of urban land, bareland and fallow land, which require the use of high-quality remote sensing imagery and seasonal consistency to minimize uncertainties (Li et al. 2017). The accuracy assessment is essential for adequately characterizing spatial data concerning land cover. In the GAA, while some classes (water, urban) exhibited high accuracy, four classes (i.e. irrigated agriculture, rainfed agriculture, shrubland, and fallow land) exhibited extremely low accuracy. This should be considered when interpreting the results.

Secondly, the utilization of solely land cover data for the analysis of ES provision has inherent limitations, including the inability to adequately reflect the potential for management options. For instance, differentiating between plantations and natural forests can be challenging in forest areas, particularly when relying solely on surface observations due to their analogous spectral characteristics (Wang et al. 2019). This makes it challenging to differentiate between them using traditional remote sensing techniques. Furthermore, although we have used imagery from the same season across different years to minimize the uncertainties that may be caused by extreme weather and cloudy data, it is essential to recognize that the land cover classification essentially captures a momentary state and does not account for ongoing changes or seasonal variations (Xie et al. 2023) that could significantly influence the accuracy and applicability of our findings. This is particularly evident in the case of water areas, which may have a high extent during certain months studied but are subject to seasonal fluctuations. Additionally, production decisions regarding agricultural land can change rapidly, with short-term transitions between pasture and other crops occurring even within a single cropping period.

To ensure the relevance of the MAES results for spatial planning, it is of the utmost importance to emphasize the significance of careful selection of ES in consultation with stakeholders. The selection of ES may be driven by pressing environmental issues (Adem Esmail and Geneletti 2017) or more generally by social challenges (Burkhard et al. 2018). In other cases, the need to cover thematically adjacent services (Nedkov and Burkhard 2012; Albert et al. 2014; Laco 2021) or a more comprehensive approach with different ES (Campagne et al. 2020; Zepp and Inostroza 2021) could be considered. This process ensures alignment with local priorities and needs, as highlighted by (Azadi et al. 2012). In this study, our deliberate selection of a wide range of ES aims to comprehensively analyze the challenges of sustainable spatial planning. In future studies, the use of surveys involving stakeholders and local experts (such as those from the Ministry of Land, Water and Environment, the Eritrean Institute of Technology, or the Hamelmalo College of Agriculture for the GAA case study) may provide more justification for the selection of specific ES.

The application of the ES matrix approach is not without its limitations, as evidenced by the self-reflective reviews of its authors (Jacobs et al. 2015; Campagne et al. 2020). A primary limitation lies in the potential mapping, which represents the maximum expression of an ES. Nevertheless, achieving this potential is contingent upon high seasons, which typically occur around August and September for the GAA. Rain-dependent agricultural and grazing land often remains fallow during other periods, resulting in post-harvest waste and potential soil degradation, including erosion, despite favorable values in the ES matrix. The assessment of the ES matrix value is also contingent on the quality of forest areas (Laco 2021) or more generally of the ecosystem conditions (Burkhard et al. 2018). Therefore, in addition to the assessment of potential ES, the exploration of the concepts of flow and demand can further enrich the assessment, providing a more comprehensive understanding of the ES dynamics.

A second limitation pertains to the fact that the ES matrix is often evaluated based on expert opinion: an approach that would benefit from corroboration by reliable local socio-ecological data. As noted by (Campagne et al. 2020), the utilization of more complex and higher-tier ES models that account for the underlying ecological structures and functions that generate the ES can be achieved by accessing better datasets or conducting new field measurements and surveys. For example, the efficacy of erosion control is primarily determined by factors such as slope and soil erodibility, including grain size and related properties, which are not considered in the ES matrix approach. The incorporation of digital elevation models (DEMs) and soil factors into the determination of erosion hazards significantly enhances the reliability of the resulting data (Adem Esmail and Geneletti 2017). Similarly, the determination of provisioning ES, such as yield per hectare, based on samples from ‘typical’ rainfed farms enables the formulation of confident ES assessments (Geneletti et al. 2018).

It is noteworthy that water represents an important provisioning and supporting ES whose value extends beyond that which can be fully captured by the ES matrix. For instance, irrigated agriculture was assigned the highest score of 5 in the ES matrix (Table 1), primarily due to the availability of water. Furthermore, most of the complex vegetation is dependent on water for nourishment. As a supporting service, water exerts a significant influence on other ES, particularly in regions where water is scarce, such as the GAA. Nevertheless, the limitations of the ES matrix in representing these water dependencies result in relatively low values that do not accurately reflect the importance of water areas.

In general, it is crucial to recognize the degree of abstraction inherent in the ES matrix approach, as exemplified by the assessment of erosion control or water ES. The ES matrix does not account for interdependencies between areas. While this level of abstraction facilitates comprehension for laypersons during participatory processes, it may not be sufficient for expert discussions and is certainly inadequate for decision-making. Consequently, the ES matrix should be regarded as a provisional approximation of reality rather than a precise representation. Supplementary and more comprehensive studies are essential to validate the ES maps and justify actions beyond these limits, thereby ensuring the legitimacy and effectiveness of conservation and management efforts in the landscape. While a proper validation of the ES maps goes beyond the scope of the present study, it indeed represents a potential avenue for future investigation and analysis.

The method applied to map hotspots and coldspots of potential ES supply has some limitations too, and any interpretation of the results must be aware of them. First, all uncertainties related to the input maps described above, from potential errors in the land cover classification to variances in ES potential not duly accounted for by the simple matrix approach, are reflected in the results of the hotspots analysis. Second, we calculated the statistic on maps of provisioning and regulating ES that only accounted for two and representative ES, respectively. Although regulating ES is often in a synergistic relationship, trade-off can also be expected, especially among different provisioning ES and between them and ES from other categories (Howe et al. 2014). Therefore, the reader must consider the specific ES selected for the analysis when interpreting the results, which also applies to the total ES maps and related changes.

In addition, as mentioned in the introduction, there are different possible interpretations of the “hotspots” and “coldspots” terms in the ES literature, which go in parallel with the methods applied to calculate them (Schröter and Remme 2016). Here, the terms refer to statistically significant spatial clusters of high and low values. It is important to note that high and low are relative to the range of values in the input map, therefore hotspots might be clusters of values that, in absolute terms, are quite low, but significantly higher than the average of the map. This is indeed the case of most of the maps in Fig. 8. Consequently, for a correct interpretation, it is important to keep in mind the range of values of the input maps. For the overall purpose of informing planning and management decisions aimed at enhancing the provision of ES in the region, combining information on the hotspots and coldspots with the total ES value is therefore crucial.

From the description above, it also follows that comparing the maps of hotspots and coldspots of different years must be done with care, especially when the range of values of the input maps is not the same or when the search radius automatically set by the tool to optimize the analysis changes. An area might cease to be a hotspot not just following a decrease in the average ES value, but also due to a mixed trend or even to an increase in specific locations that makes the clustering not significant anymore. Therefore, the results of the hotspots and coldspots analysis, especially when comparing multiple time steps, should always be interpreted in combination with the maps that were used as inputs.

### Insight for Sustainable Spatial Planning in the Greater Asmara Area

Despite limitations such as low values in accuracy assessment of the land cover mapping and ES matrix based on secondary data, the study provides a comprehensive overview of ES potentials within the GAA and their changes over the past decade. In particular, the hotspots and coldspots dynamics offer valuable insight for sustainable planning and policymaking. Given the scarcity of studies on ES in this area, this research holds significant importance. The findings can contribute to the raising of awareness of the direct and indirect benefits humans gain from ecosystems.

Enhancing the potential of provisioning ES is key for the achievement of food security objectives in Asmara and Eritrea as a whole. Nationally, agriculture provides employment for up to 80% (Ghebru et al. 2011; Hunt et al. 2019) of the population, with many relying on subsistence farming. This reliance is evident also in the study area, although the GAA has also important industrial and service sectors (Ministry of Public Works and BCEOM 2006). The assessment of provisioning ES in the GAA reveals a stable trend (+0.15) in the potential for *ES1—Cultivated terrestrial plants grown for nutritional purposes*. For the potential of *ES2—Animals reared for nutritional purposes*, the overall trend in the GAA remained relatively stable, with slight increases in Gala Nefhi.

The observed trends in the potential of *ES1* and *ES2* can be related to the agriculture shifts evidenced by our analysis, with increased irrigated farming in Gala Nefhi and Northern Mereb, and declines in rainfed agriculture in Northern Asmara and Southern Mereb. Specifically, the increase in the potential of *ES1 - Cultivated terrestrial plants grown for nutritional purposes* underscores the importance of supporting agricultural development in these regions, particularly through irrigated agriculture. It is therefore recommended that sustainable practices and land and water resource management be emphasized to maintain and enhance these gains. The stable trend in the potential of *ES2—Animals reared for nutritional purposes* suggests a balanced approach to grazing land management in the GAA. However, targeted interventions in regions showing significant growth, such as Gala Nefhi, can further enhance grazing potential and contribute to food security. Indeed, modern pastoralism is believed to possess the advantage of being relatively resilient to droughts, as noted by Sinare et al. (2016). Nevertheless, it is crucial to consider both the opportunities and risks associated with modern pastoralism, as discussed, for example, by previous studies by Weber and Horst (2011). Further disaggregated assessment is also required to ascertain the costs and benefits associated with ES, particularly for the poor (Daw et al. 2011). Among others, this should consider the complex chain between the “Services Providing Units” (SPU) and the “Services Benefiting Area” (SBA), i.e. the areas that provide the provisioning service and the area where beneficiaries are located (Burkhard et al. 2014).

The assessment revealed noteworthy trends in the potential of regulating services in the GAA. Firstly, the potential of *ES3 - Control of water erosion rates* show an overall improvement across different sub-regions, with Gala Nefhi experiencing a significant increase. Conversely, the potential of *ES4 - Regulation of temperature and humidity* demonstrates a stable trend, with Gala Nefhi exhibiting a moderate increase. The potential for *ES5 - Maintaining or regulating nursery populations and habitats* has improved across various sub-regions, particularly in Gala Nefhi, while Northern Asmara experienced a stable trend. These dynamic and nuanced patterns of change of the regulating ES potential emphasize the importance of targeted interventions to maintain and enhance these vital ES in the GAA.

In this regard, increasing vegetation cover, whether dense (e.g., forest) or semi-open (e.g., rainfed, irrigated, shrubland), plays a crucial role for enhancing the potential of several regulating ES. On the contrary, (Wangai et al. 2019) have underscored, among other ES functions, the adverse impact of urbanization on microclimate regulation in African cities. In the context of the ES matrix, a transition from forest to urban areas results in a decline from a potential score of 4 to 0 or 1 for other land types. In addition, it’s important to recognize that earmarking some bareland areas for development to preserve higher-value land and promoting densification to reduce urban footprint on the landscape—as done by the SUDP—can be an equally effective approach alongside interventions to enhance bare land.

Indeed, signs of positive development are observable in the GAA, notably in the emergence of newly created shrub lands, which may eventually evolve into forested lands. The results highlight significant urbanization in Berik and Northern Mereb, with forested areas showing steady growth in Gala Nefhi, South Mereb, and South Asmara. This is quite in line with previous analysis at the national level (Adem Esmail et al. 2023). Eritrea has demonstrated its commitment to afforestation and desertification combat through the identification of afforestation areas in the SUDP (Ministry of Public Works and BCEOM 2006; MoLWE 2012b). However, the success of an afforestation strategy is dependent on access to water, particularly during the vulnerable initial stages. The afforestation and plantation efforts mentioned, like most forested areas, are concentrated around water sources in the northern subzones in the GAA.

The assessment of the cultural ES within the GAA reveals a rather low potential, with a mean value of 0.8 in 2009 and a stable trend (+0.03 in 2020). It is safe to conclude that this assessment aligns with the ‘lived experience’ of the residents, particularly in the Asmara proper. For example, except for a few urban parks, residential structures in Asmara lack forests or other forms of landscaping, with a disproportionate impact on marginalized settlements in the central parts of the city proper. This ‘lack’ is echoed at the national level, where the forest coverage is between 1 and 2% (Ghebru et al. 2011). Therefore, considering the significant physical and mental health benefits (Kabisch et al. 2017), restoring the connection of people in cities with nature is indeed a key challenge that must be considered seriously (Andersson et al. 2014).

It is evident that urban planning has the potential to facilitate the greening of cities to address multiple societal challenges (Adem Esmail et al. 2022). It is therefore commendable that reforestation was a key strategy advocated for the GAA, already twenty years ago by the SUDP (Ministry of Public Works and BCEOM 2006). Besides valuable agricultural land (Tesfagiorgis 2004), the SUDP prioritizes the preservation of natural areas in marginal settlements, which are not bounded by urban structures. This strategic approach has the potential to enhance the cityscape of Asmara and contribute to its overall appeal. However, beyond planning initiatives, it is essential to allocate adequate resources, establish robust institutions, and actively involve citizens in environmental conservation efforts to ensure sustainable development and long-term success. Awareness programs with citizens taking agency in greening their neighborhoods/residential sites, with children being culturalized into this through school program, and adequate incentives from local authorities are some of the steps that need consideration. From a research perspective, almost 25 years after its approval, the SUDP offers an insightful case study to explore what and how determines the implementation gap of strategic urban planning in Global South contexts (Li et al. 2022).

Hotspots coldspot analysis illustrates that different ES dynamics are at play in different parts of the GAA. Understanding some of the implications for sustainable spatial planning, however, requires carefully accounting for context-specific considerations. For instance, in Northern Mereb, the disappearance of coldspots and the appearance of new hotspots between 2009 and 2020 corresponds to a significant increase in the water area and larger irrigated areas. In contrast, the Mai Bela floodplain in Berik saw its hotspots nearly disappear by 2020 may be due to reduced precipitation, shifts in land cover, and related changes in the ES potential. Again, in Southern Mereb/Gala Nefhi—Asmara Airport, the appearance of a coldspot in the central part of Gala Nefhi can be linked to the “Asmara Ring Road Project”: a major highway built in 2017 to divert heavy traffic away from the capital (Shabait 8 Feb 2017, https://shabait.com/2017/02/08/ring-road-project/, accessed 05 June 2024). These examples in the GAA provide insight into how analysis of hotspots and coldspots dynamics can inform and support sustainable spatial planning, indicating areas that require protection (hotspots) and ecological restoration (coldspots) to meet diverse needs. However, these should be interpreted cautiously, as they are influenced by several factors, including the number of features, value ranges, and optimal search distance. While maps can effectively compare different areas within the same year, inter-year comparisons require additional expert analysis or ground truthing. A qualitative comparison of four illustrative areas in the GAA with significant changes in terms of hotspots and coldspots distribution between 2009 and 2020 is presented in Fig. A13 in the SM.

## Conclusion

This study demonstrates that mapping and analyzing ES hotspots and coldspots dynamics provides valuable insights for sustainable spatial planning in rapidly urbanizing African metropolitan regions like the GAA. The ES matrix approach, based on remote sensing data, offers a cost-effective way to map and assess ES dynamics, identify key regions for further investigation, and implement ecosystem protection or enhancement interventions. Despite some limitations, five key conclusions from this MAES pilot in the GAA are:

- Recent changes in land cover in the GAA have favored provisioning ES, particularly with the expansion of irrigated areas.
- Although afforestation has yet to show clear positive results in forest areas, the potential development in shrub areas mapped for 2020 highlights positive expectations and the need for ongoing ES monitoring.
- Overall ES potential in the GAA remains low but stable, with some improvements. By 2020, areas with no ES potential decreased in southern regions like Gala Nefhi and Berik, and coldspots in Northern Mereb decreased, with new hotspots and coldspots emerging in central Gala Nefhi.
- Integrating the ES perspective, especially aligned with the UN Agenda 2030, is essential to prevent degradation from land cover changes, ensure food and water security, and enhance resilience in the GAA. The upcoming renewal of the Strategic Urban Development Plan for the GAA, expiring in 2025, offers a key opportunity to advance and mainstream the ES approach in sustainable spatial planning.

## Supplementary Information

Supplementary Material

## Data Availability

Data are provided within the manuscript or supplementary information files.
