# Supplementary material for: Mapping and Analyzing Ecosystem Services Hotspots and Coldspots for Sustainable Spatial Planning in the Greater Asmara Area, Eritrea
Source: Environ Manage. 2024 Nov 3;75(3):551–67. doi: 10.1007/s00267-024-02078-x (PMC11861412; doi:10.1007/s00267-024-02078-x)
Supplement: Supplementary file 1 — Supplementary Material [file 267_2024_2078_MOESM1_ESM.docx]

# Supplementary Material

## Section A - Land cover change analysis

### **A1. Accuracy assessment**

Table A 1. Accuracy Assessment Values for the 2009 and 2020 classification

|  | 2009 | | 2020 | |
| --- | --- | --- | --- | --- |
|  | Producer Accuracy | Consumer Accuracy | Producer Accuracy | Consumer Accuracy |
| Water | 1 | 1 | 1 | 0.96 |
| Forest | 1 | 0.8 | 0.67 | 0.67 |
| Irrigated agr | 0.75 | 0.67 | 0.33 | 0.5 |
| Rainfed agr | 0.67 | 0.67 | 0 | 0 |
| Shrubland | 0.54 | 0.6 | 0.2 | 0.5 |
| Fallow Land | 0.5 | 0.5 | 0.33 | 0.4 |
| Urban | 0.75 | 0.75 | 1 | 1 |
| Grazing Land | 0.6 | 0.75 | 0.8 | 0.8 |
| Overall Accuracy | 0.79 | | 0.75 | |
| Kappa Value | 0.74 | | 0.67 | |

### **A2. Land cover data**


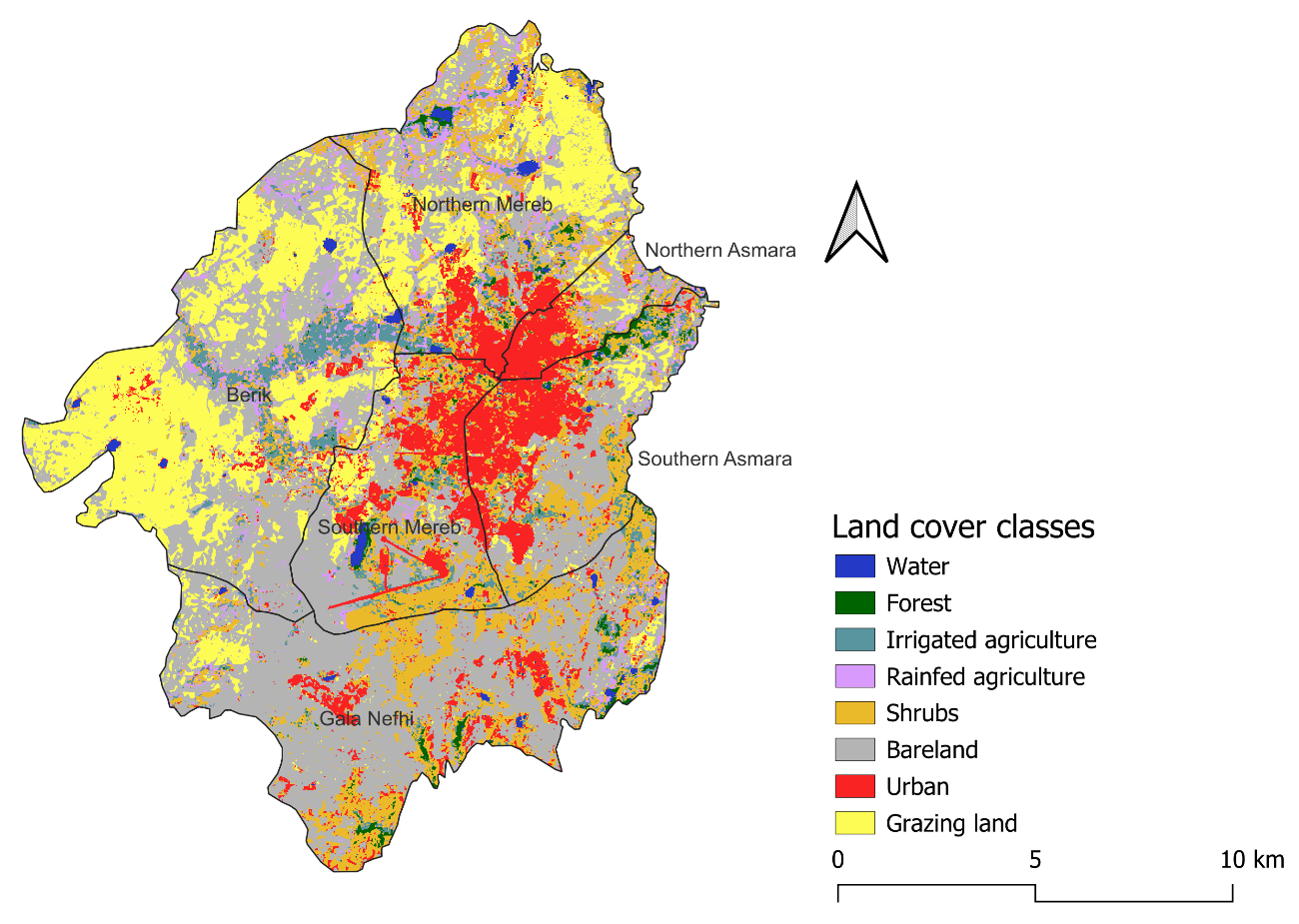


Figure A 1. Land cover classification in 2009.


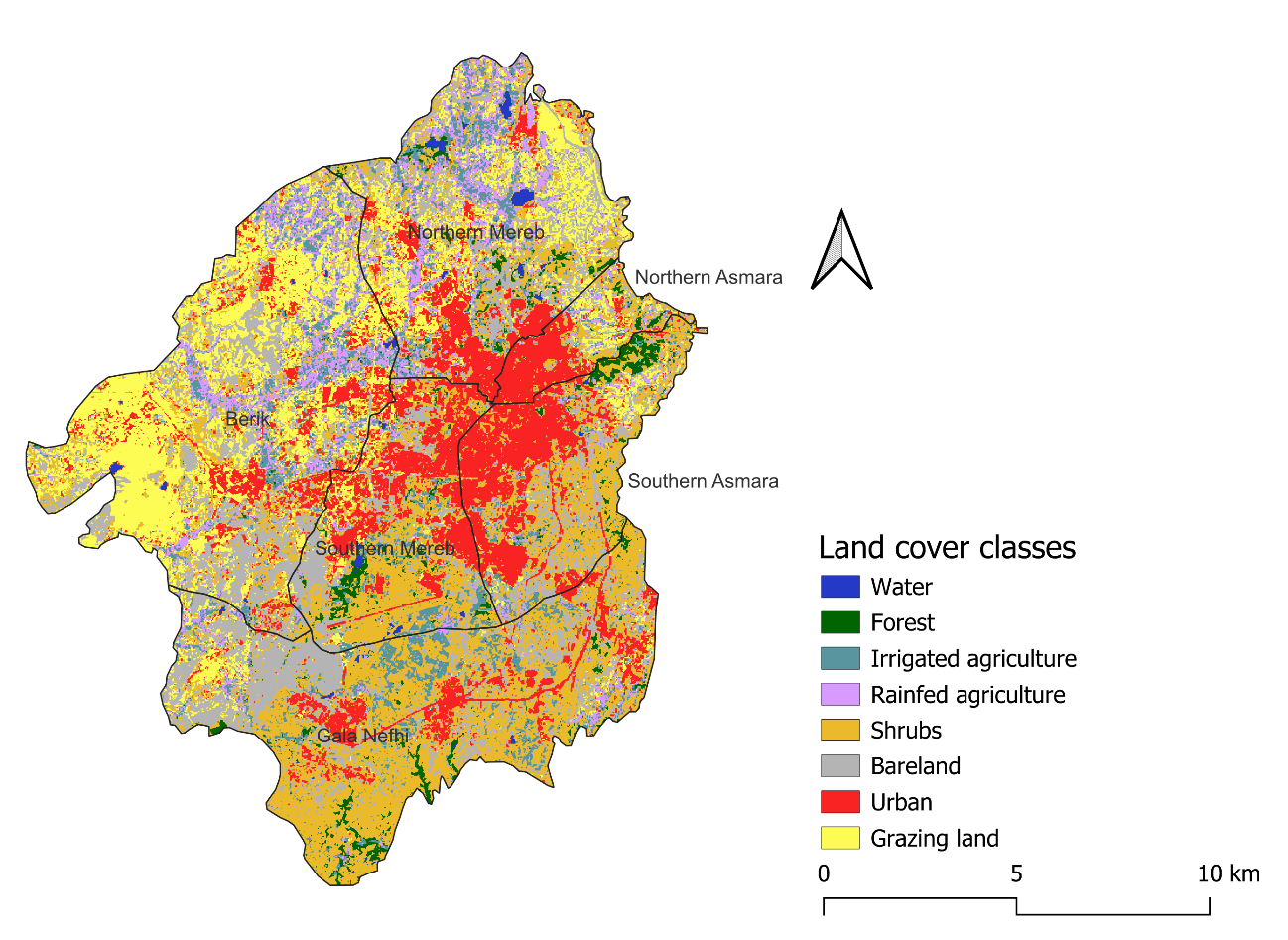


Figure A 2. Land cover classification in 2020.


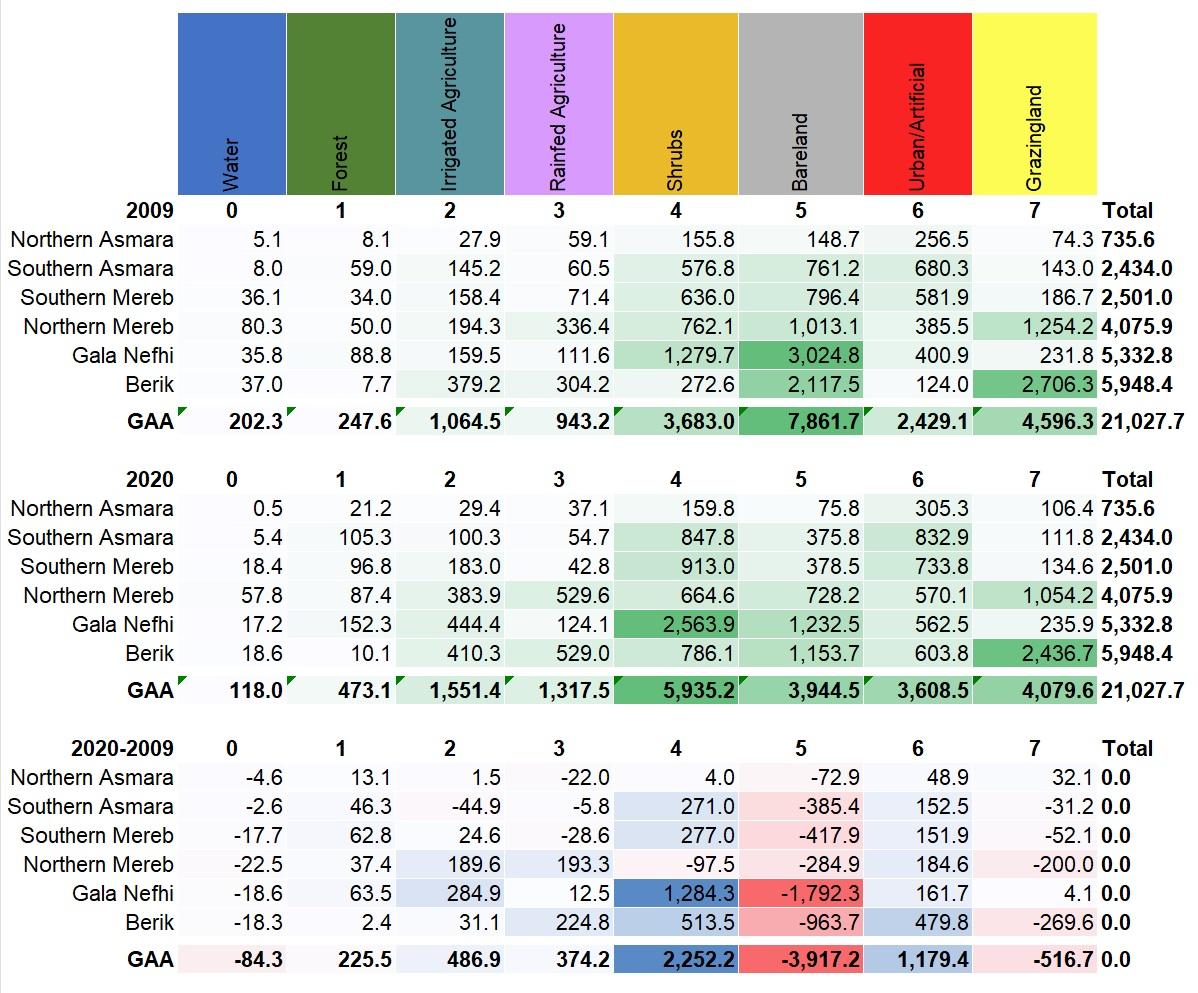


Figure A 3. Land cover Land cover distribution and changes in absolute terms (ha) between 2009 and 2020.


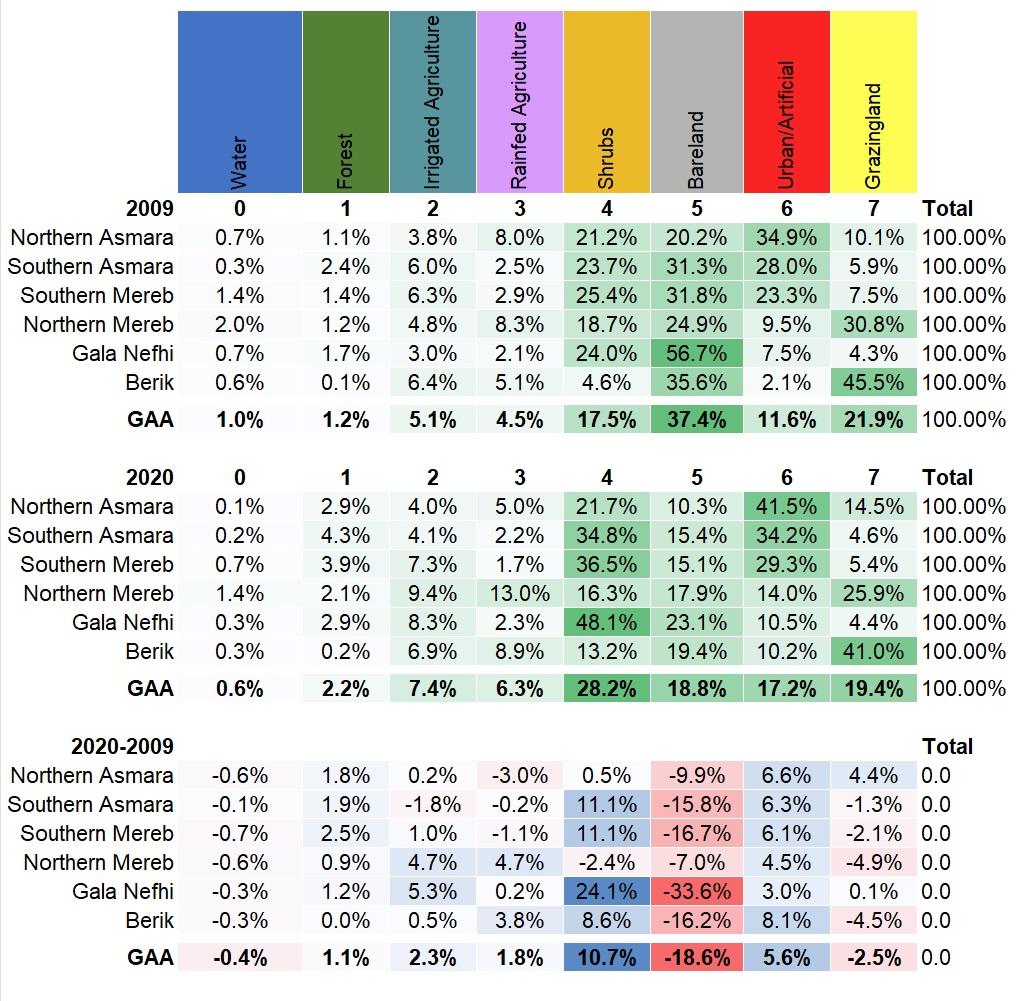


Figure A 4. Land cover distribution and changes in percentage (%) between 2009 and 2020.


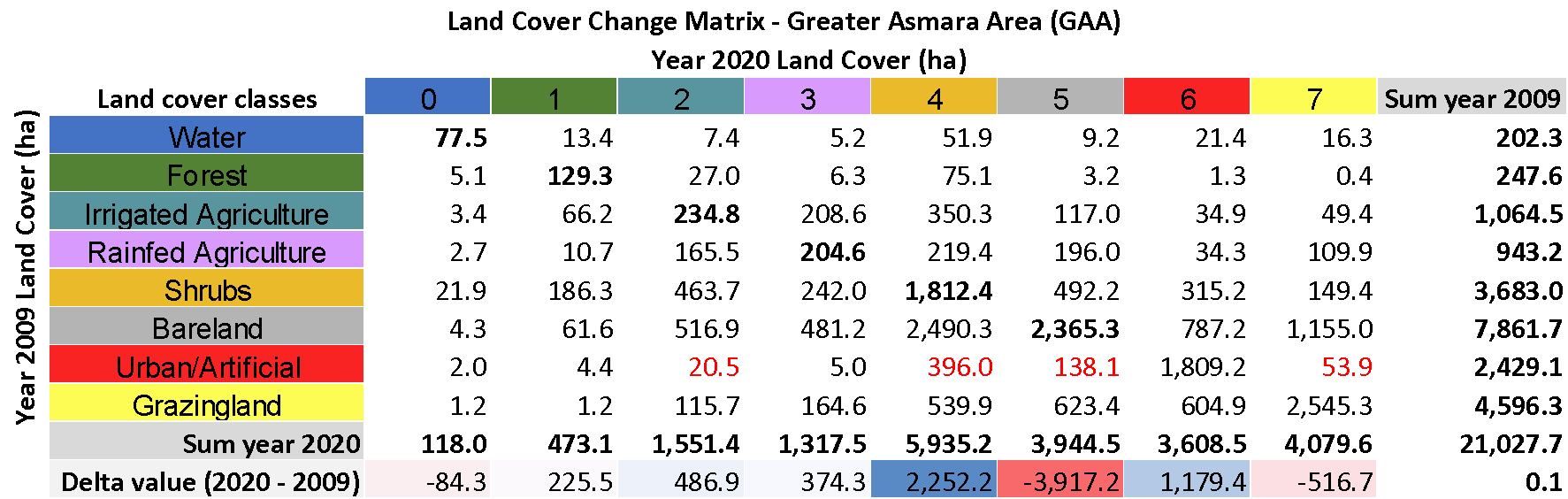


Figure A 5. Land cover transition matrix between 2009 and 2020): it describes the conversion of land cover classes between the initial year 2009 and final year 2020. Each row represents a land cover class in the initial year, and each column represents the amount of conversion to the new land cover class in the final year.

## Section B - Mapping and assessment of ES

### **B1. ES potential maps for 2009 and 2020**


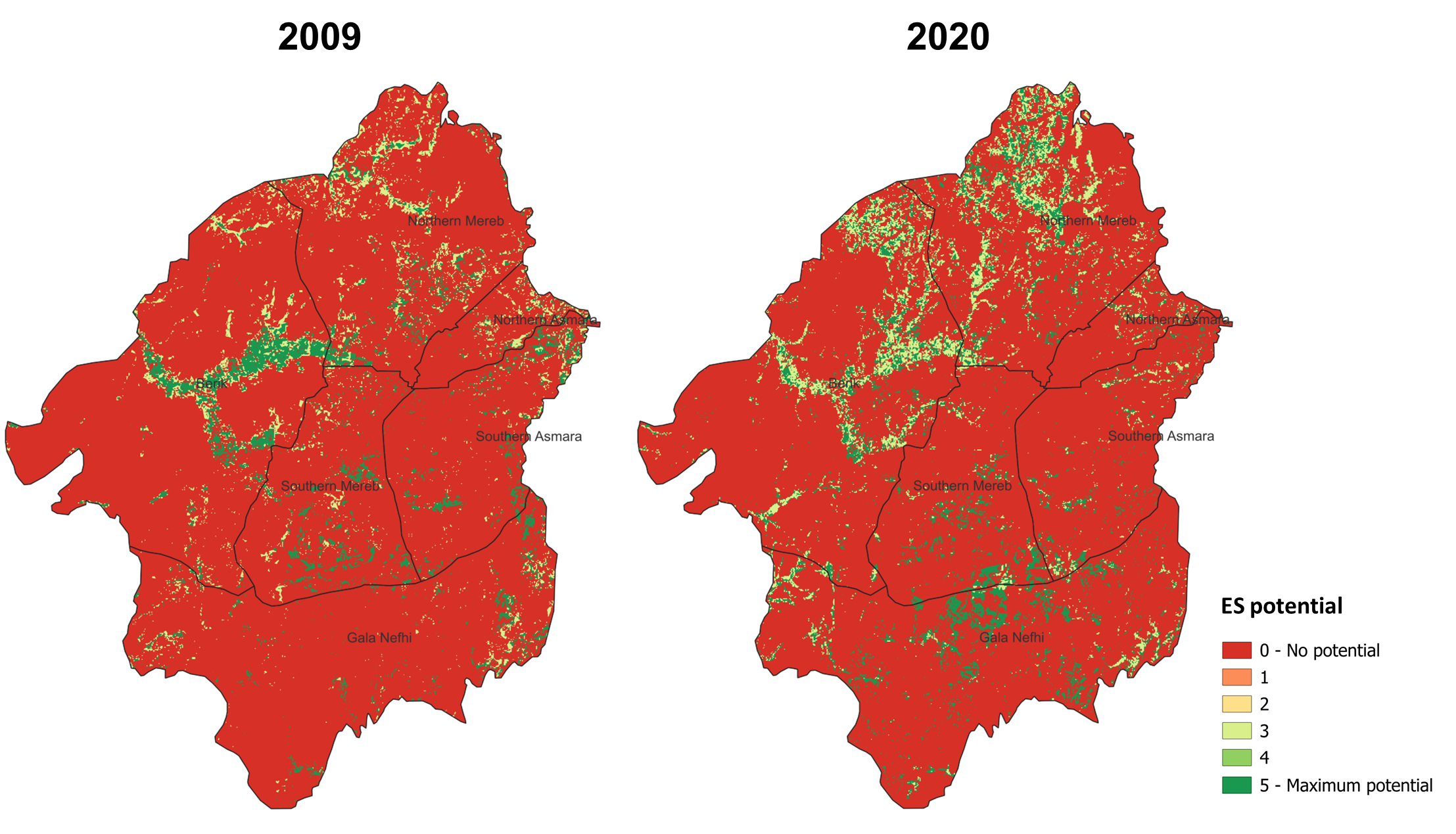


Figure A 6. Mapping potential of ES1 - Cultivated terrestrial plants grown for nutritional purpose in 2009 and 2020.


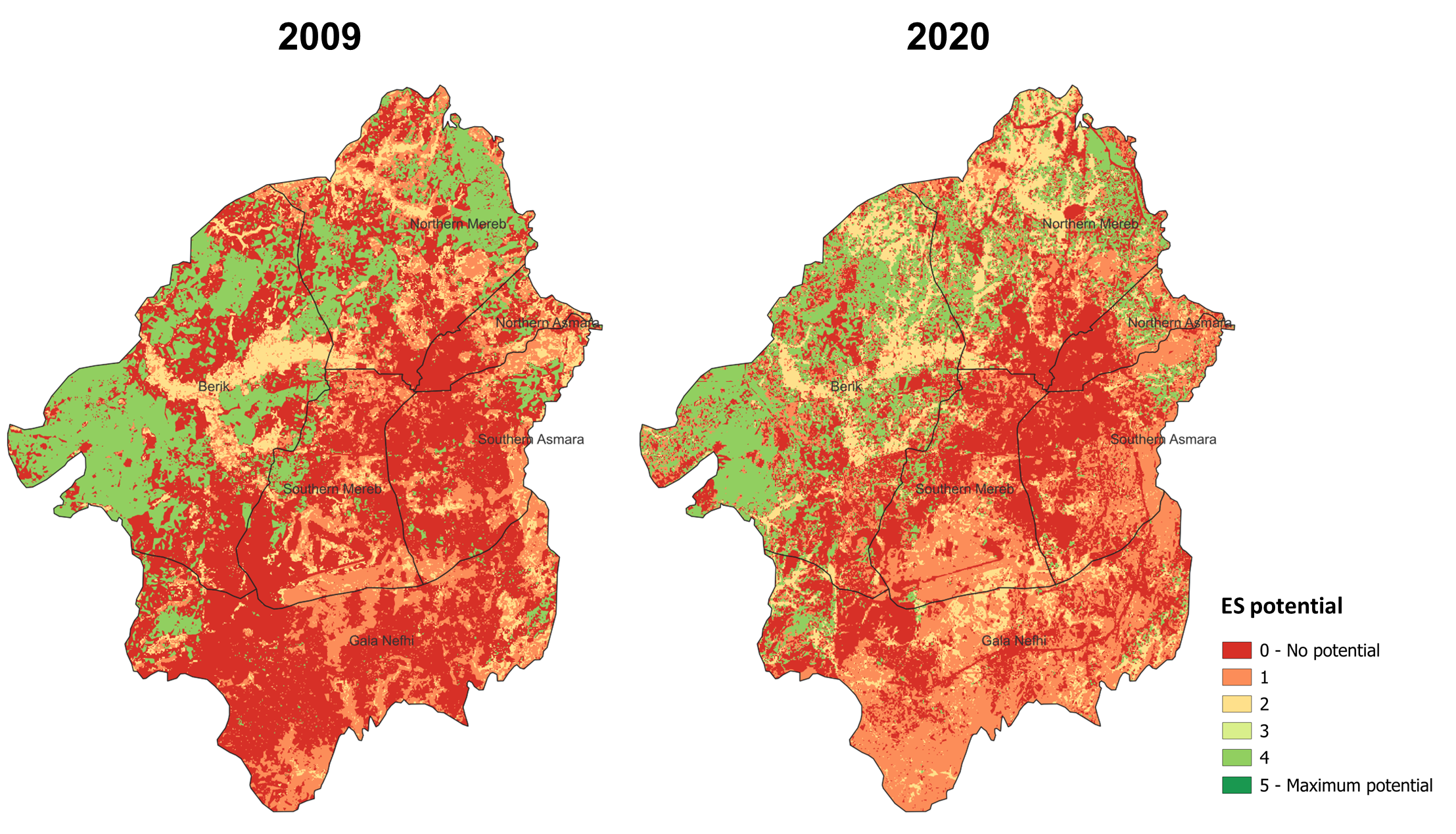


Figure A 7. Mapping potential of ES2 - Animals reared for nutritional purposes in 2009 and 2020.


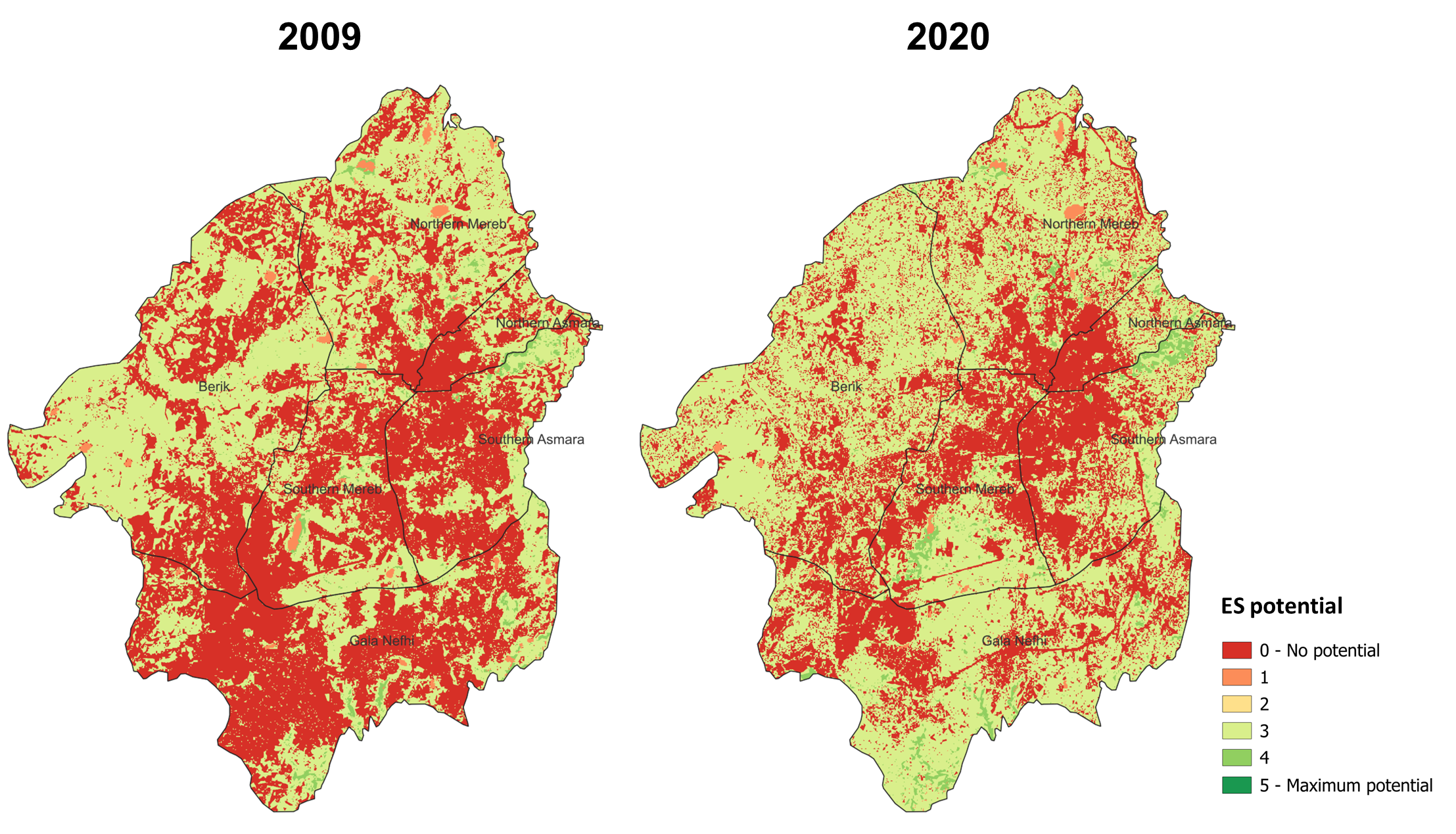


Figure A 8. Mapping potential of ES3 - Control of erosion rates in 2009 and 2020.


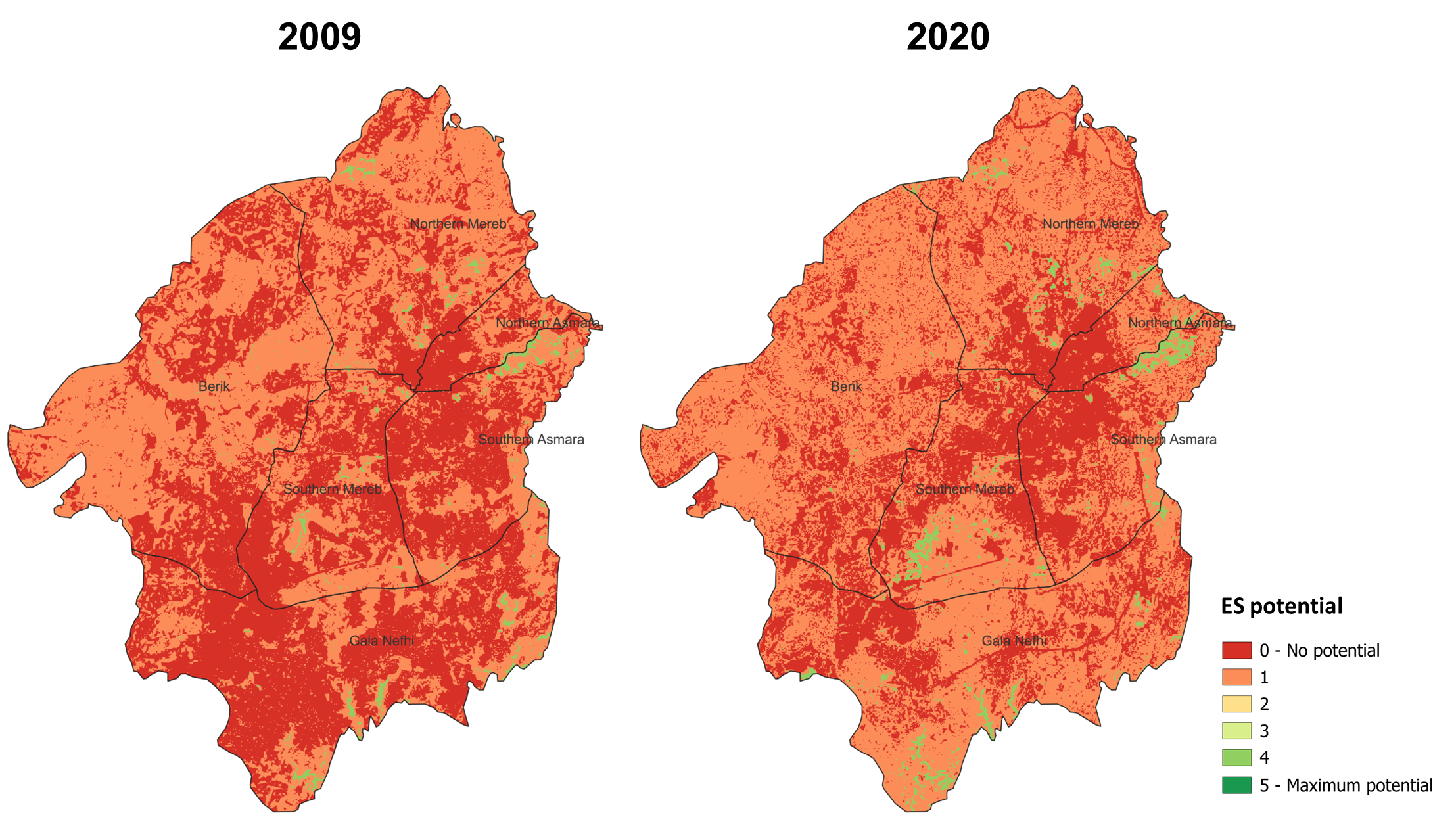


Figure A 9. Mapping potential of ES4 - Regulation of temperature and humidity, including ventilation and transpiration in 2009 and 2020.

*
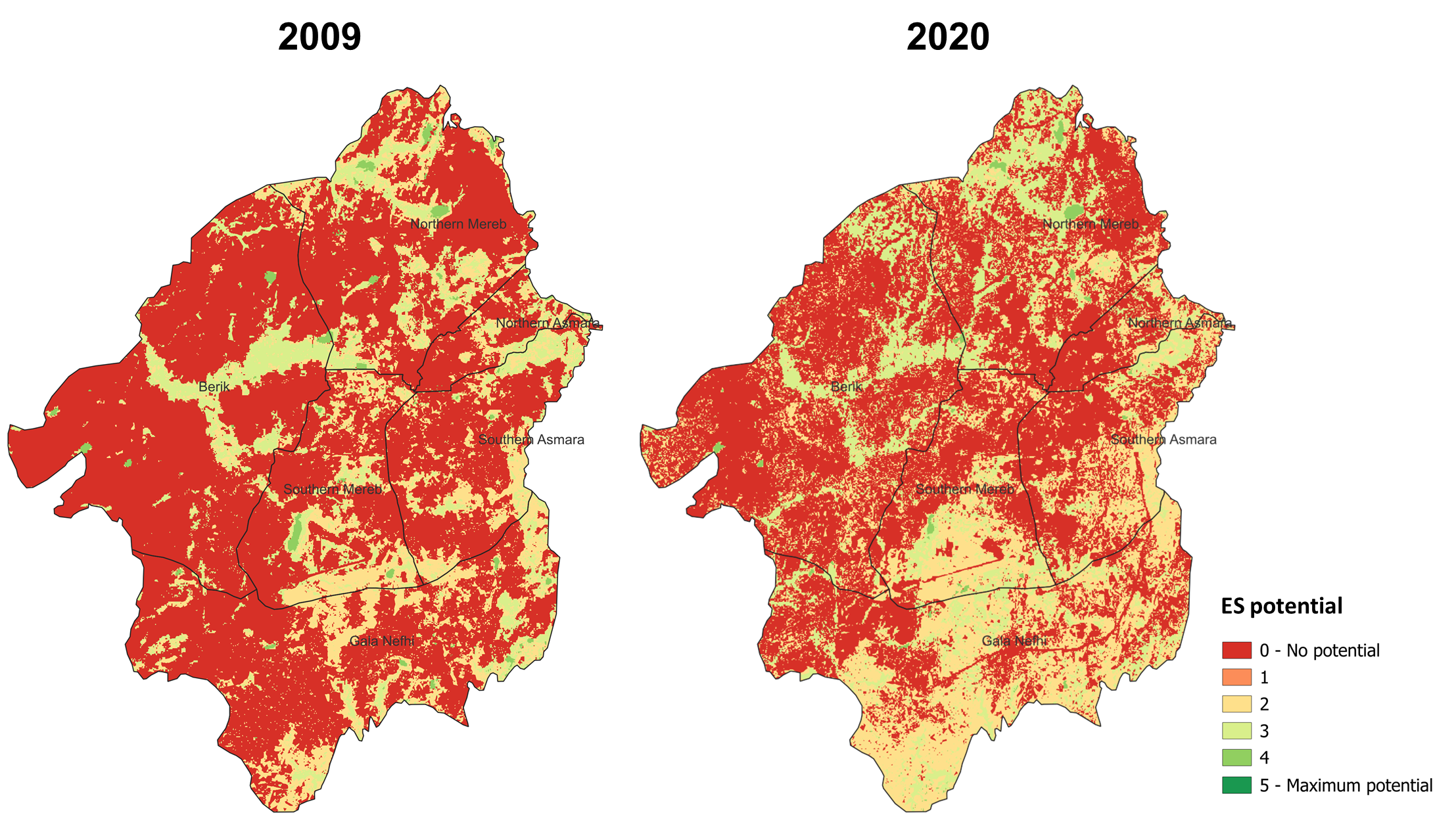
*

Figure A 10. Mapping potential of ES5 - Maintaining nursery populations and habitats in 2009 and 2020.


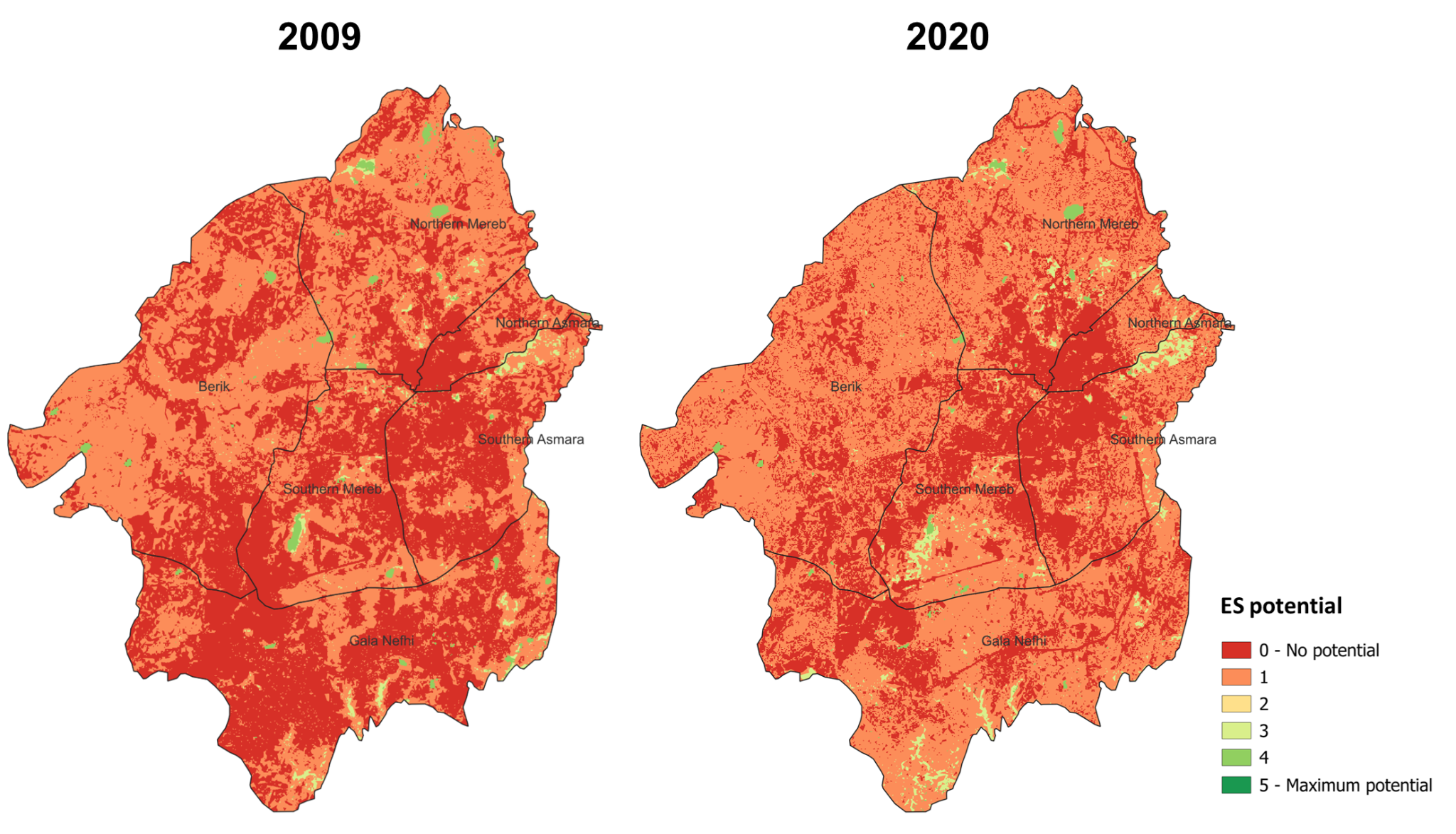


Figure A 11. Mapping potential of ES6 - Characteristics of living systems that enable activities promoting health recuperation or enjoyment through active or immersive interactions in 2009 and 2020.


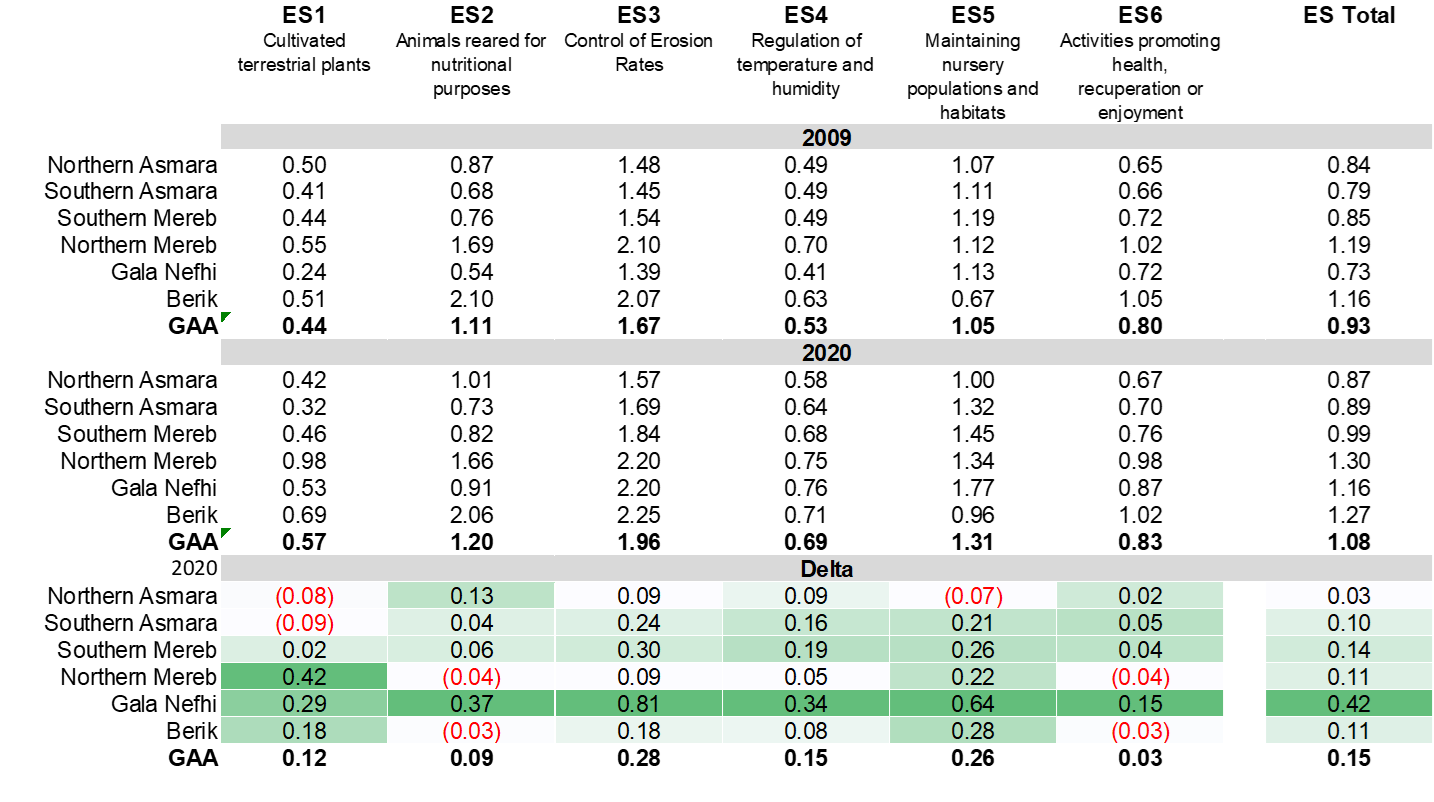


Figure A 12. Changes in the mean value of the ES potential in the GAA subzones between 2009 and 2020.

## Section C - Hotspot and cold spot analysis results


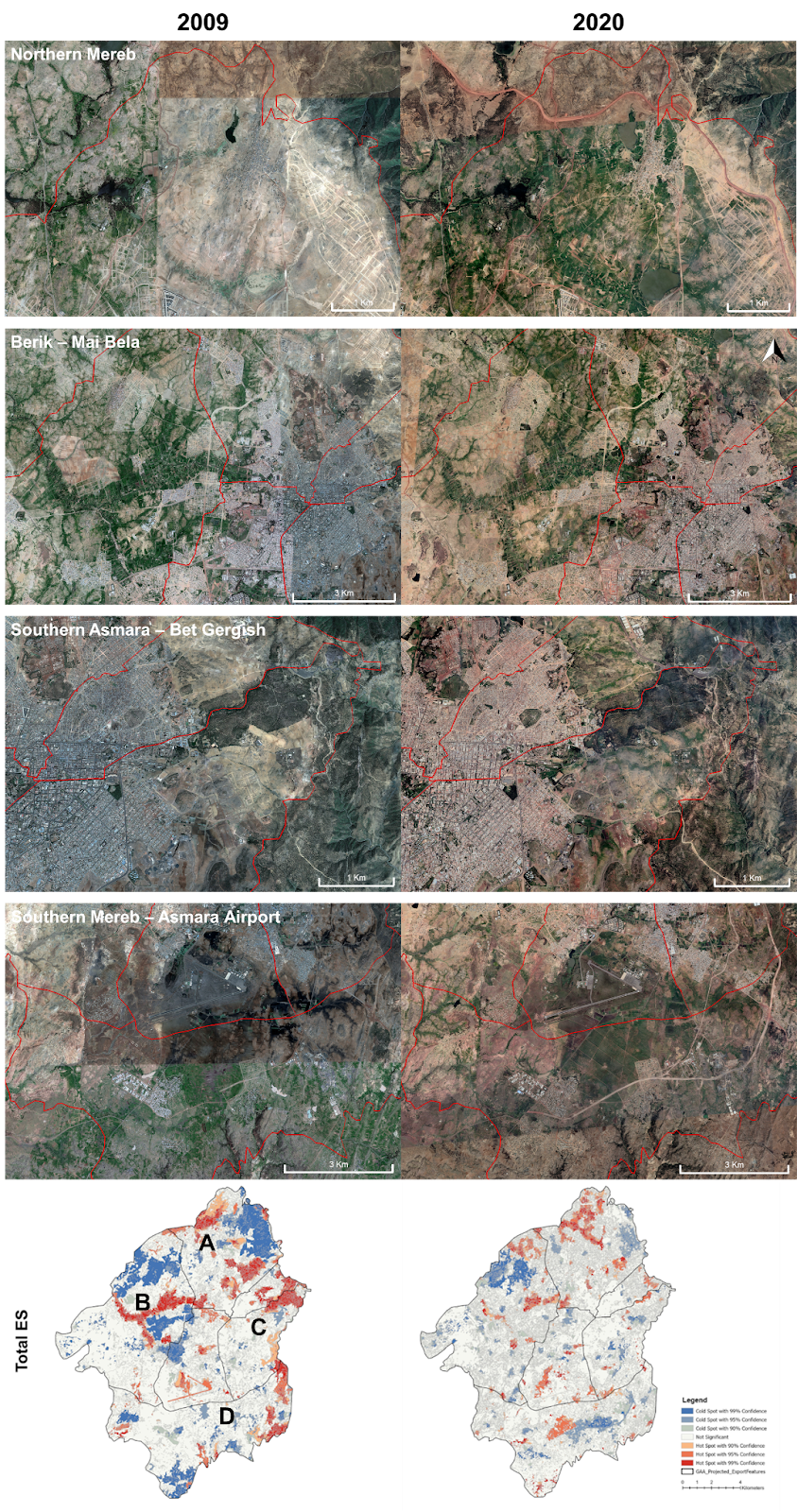
Figure A 13. Qualitative comparison of four illustrative areas in the GAA with significant changes in terms of hotspots and cold spots distribution between 2009 and 2020. Area A (Northern Mereb): Significant increase in the water area and hotspot growth from 2009 to 2020. Area B (Berik - Mai Bela floodplain): Nearly disappeared hotspots by 2020, with Google Earth images showing a drier landscape and reduced irrigated areas. Area C (Southern Asmara - Betgergish): Hotspot within the Bet Gergish protected area vanished in 2020, with no clear explanation from Google Earth images. Area D (Southern Mereb/Gala Nefhi - Asmara Airport): A cold spot in Gala Nefhi linked to the Asmara Ring Road Project (2017). Hotspot south of the airport is related to more cultivated land and conversion into shrubland, as observed in land cover analysis. Disappearing hotspot along the eastern border of Gala Nefhi with no clear change except a drier landscape. (Source: Google Earth image © 2024 Maxar Technologies)
